# Animal models of Duchenne muscular dystrophy: from basic mechanisms to gene therapy

**DOI:** 10.1242/dmm.018424

**Published:** 2015-03

**Authors:** Joe W. McGreevy, Chady H. Hakim, Mark A. McIntosh, Dongsheng Duan

**Affiliations:** 1 Department of Molecular Microbiology and Immunology, School of Medicine, University of Missouri, Columbia, MO 65212, USA; 2 Department of Neurology, School of Medicine, University of Missouri, Columbia, MO 65212, USA

**Keywords:** Duchenne muscular dystrophy, Dystrophin, Animal model, Canine DMD, Gene therapy

## Abstract

Duchenne muscular dystrophy (DMD) is a progressive muscle-wasting disorder. It is caused by loss-of-function mutations in the dystrophin gene. Currently, there is no cure. A highly promising therapeutic strategy is to replace or repair the defective dystrophin gene by gene therapy. Numerous animal models of DMD have been developed over the last 30 years, ranging from invertebrate to large mammalian models. *mdx* mice are the most commonly employed models in DMD research and have been used to lay the groundwork for DMD gene therapy. After ~30 years of development, the field has reached the stage at which the results in *mdx* mice can be validated and scaled-up in symptomatic large animals. The canine DMD (cDMD) model will be excellent for these studies. In this article, we review the animal models for DMD, the pros and cons of each model system, and the history and progress of preclinical DMD gene therapy research in the animal models. We also discuss the current and emerging challenges in this field and ways to address these challenges using animal models, in particular cDMD dogs.

## Introduction

**Fig. 1.**
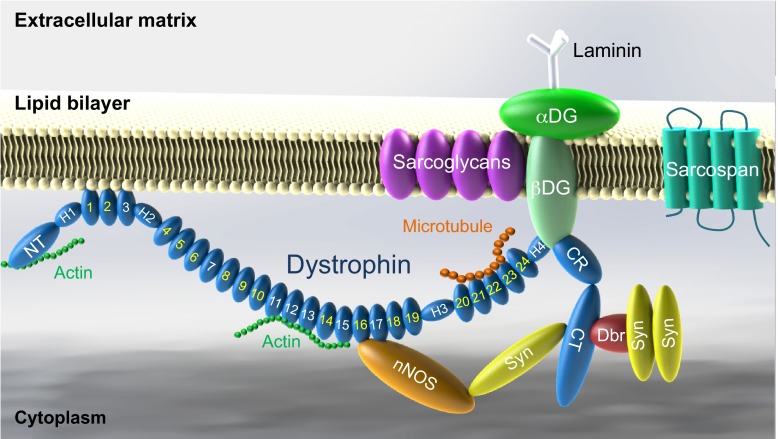
**Schematic outline of dystrophin and the dystrophin-associated glycoprotein complex (DAGC).** Dystrophin contains N-terminal (NT), middle rod, cysteine-rich (CR) and C-terminal (CT) domains. The middle rod domain is composed of 24 spectrin-like repeats (numerical numbers in the cartoon, positively charged repeats are marked in white color) and four hinges (H1, H2, H3 and H4). Dystrophin has two actin-binding domains located at NT and repeats 11–15, respectively. Repeats 1–3 interact with the negatively charged lipid bilayer. Repeats 16 and 17 form the neuronal nitric oxide synthase (nNOS)-binding domain. Dystrophin interacts with microtubule through repeats 20–23. Part of H4 and the CR domain bind to the β-subunit of dystroglycan (βDG). The CT domain of dystrophin interacts with syntrophin (Syn) and dystrobrevin (Dbr). Dystrophin links components of the cytoskeleton (actin and microtubule) to laminin in the extracellular matrix. Sarcoglycans and sarcospan do not interact with dystrophin directly but they strengthen the entire DAGC, which consists of dystrophin, DG, sarcoglycans, sarcospan, Syn, Dbr and nNOS.

**Fig. 2.**
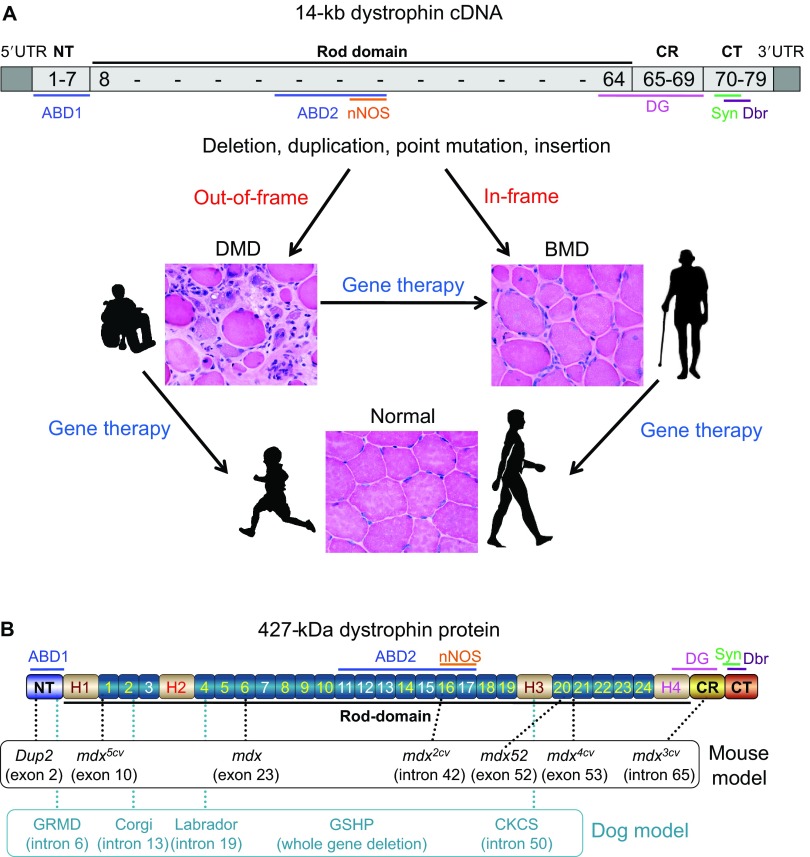
**DMD gene therapy and dystrophin mutations in animal models.** (A) The 14-kb dystrophin cDNA and the principle of DMD gene therapy. The numbers in the cDNA refers to exon number. The DNA sequence position of the main dystrophin domains and of the dystrophin-associated protein-binding sites (see Fig. 1) is also shown. Frame-interrupting (out-of-frame) mutation leads to severe DMD. In-frame mutation results in mild Becker muscular dystrophy (BMD). The primary goal of DMD gene therapy is to ameliorate muscle pathology and to improve muscle function. Gene therapy can convert the DMD phenotype to the benign BMD phenotype. Gene therapy might also prevent or slow down the development of muscle disease if affected individuals are treated early enough. (B) Domain structure of dystrophin and location of the mutations in representative mouse and dog models. ABD, actin-binding domain; CKCS, Cavalier King Charles spaniel; CR, cysteine-rich domain; CT, C-terminal domain; Dbr, dystrobrevin; DG, dystroglycan; GRMD, golden retriever muscular dystrophy; GSHP, German shorthaired pointer; nNOS, neuronal nitric oxide synthase; NT, N-terminal domain; Syn, syntrophin; UTR, untranslated region. See supplementary material Table S1 for a description on each model.

Duchenne muscular dystrophy (DMD) is the most common muscular dystrophy, with a worldwide incidence of one in 5000 live male births according to newborn screening (Emery and Muntoni, 2003; Mendell and Lloyd-Puryear, 2013). It is caused by the lack of dystrophin, a critical muscle protein that connects the cytoskeleton and the extracellular matrix (ECM) (Bonilla et al., 1988; Hoffman et al., 1987). The 2.4-Mb dystrophin gene was discovered in 1986 (Kunkel, 2005; Monaco et al., 1986). It contains 79 exons and encodes a ~14-kb cDNA (Koenig et al., 1987). The full-length protein has four functional domains: the N-terminal (NT), rod, cysteine-rich (CR) and C-terminal (CT) domains. Dystrophin assembles several transmembrane (dystroglycan, sarcoglycan, sarcospan) and cytosolic [syntrophin, dystrobrevin and neuronal nitric oxide synthase (nNOS)] proteins into a dystrophin-associated glycoprotein complex (DAGC) at the sarcolemma (Fig. 1; Box 1 for a glossary of terms) (Ervasti, 2007). Frame-shift mutations of the dystrophin gene abolish protein expression and lead to DMD (Box 1). In-frame deletions often generate truncated dystrophin and result in the milder Becker muscular dystrophy (BMD) (Fig. 2A) (Beggs et al., 1991; Hoffman and Kunkel, 1989; Monaco et al., 1988).

**Box 1. Glossary**

Adeno-associated virus (AAV): a single-stranded DNA virus identified in 1965. AAV has a ~4.7-kb genome and encodes at least three open reading frames (ORFs), one for viral capsid proteins, one for replication proteins and a third one for the assembly-activating protein. In recombinant AAV vectors, viral ORFs are replaced by a reporter or therapeutic expression cassette. An up to 5-kb vector genome can be packaged in an AAV vector. At least 13 different AAV serotypes have been reported. Hundreds of genetically modified AAV capsids have also been developed. AAV can efficiently transduce post-mitotic tissues and wild-type AAV does not cause human disease. Because of these features, AAV has been used in numerous clinical trials.
Dual and tri-AAV vectors: engineered AAV vector systems that can deliver a 10-kb (dual vector) or 15-kb (tri-vector) expression cassette. Specifically, a large expression cassette is divided into two pieces (dual vectors) or three pieces (tri-vector). An individual piece contains either a region that overlaps with another piece and/or is engineered with splicing signals. Each piece is packaged in a single viral particle. Co-delivery of vectors containing different pieces of the expression cassette results in reconstitution of the original expression cassette *in vivo* by cellular recombination mechanisms.
Exon skipping: a phenomenon in which one or multiple exons are spliced out and eliminated from the mature mRNA.
Frame-shift mutation: a mutation that disrupts the open reading frame of an mRNA transcript.
Freezing response: a reflex defense mechanism observed in prey animals where they freeze or completely stop moving when scared.
Hydrodynamic intravascular delivery: a technique used for gene delivery where the hydrostatic pressure is applied to increase the permeability of the vascular wall. This allows efficient penetration of gene therapy plasmids into the tissue parenchyma.
Liposome: an artificially created lipid-bilayer sphere. A DNA plasmid can be incorporated inside the lipid sphere. The fusion of the lipid bilayer with cell membrane allows delivery of the DNA plasmid into a cell.
Microspheres: generic name given to a nanoscale spherical object that can be made out of a variety of materials, including lipids, polymers and metal oxides. They can be used to deliver a DNA plasmid to the cell.
Nuclease-based gene editing: DNA gene editing technique that uses endonucleases to make double-stranded breaks in the DNA at a user-specified location to initiate error-prone DNA repair. As a consequence, the DNA sequence at the site of break is altered. These endonucleases are often linked to sequence-specific targeting proteins, such as zinc fingers.
Phosphorodiamidate morpholino oligomer (PMO): a synthetic oligonucleotide in which the ribose or deoxyribose backbone is replaced by a morpholine ring and the phosphate replaced by phosphorodiamidate. Any one of the four nucleobases can be attached to the morpholine ring. Because of the unnatural backbone, PMO is more resistant than the ordinary antisense oligonucleotide (AON) to nuclease digestion.
Revertant fibers: rarely occurring dystrophin-positive myofibers found in animals that carry a null mutation in the dystrophin gene. The molecular mechanisms underlying the formation of revertant fibers are not completely clear. They might arise from sporadic alternative splicing that eliminates the mutation from the dystrophin transcript and/or a second mutation that corrects the original mutation on the DNA.
Sarcolemma: muscle-cell plasma membrane.
Vivo-morpholino: a morpholino oligomer that has been covalently linked to an octa-guanidine dendrimer moiety. Conjugation with octa-guanidine increases cell penetration.
WW domain: a protein module of approximately 40 amino acids. It contains two preserved tryptophan (W) residues that are spaced 20 to 22 amino acids apart. The WW domain folds into a stable, triple-stranded β-sheet and mediates protein-protein interaction.

The identification of the disease-causing gene and the molecular basis for the DMD and BMD phenotypes establishes the foundation for DMD gene therapy (Fig. 2A). To mitigate muscle disease, one can either restore the full-length transcript or express a truncated but in-frame dystrophin gene (Duan, 2011; Goyenvalle et al., 2011; Konieczny et al., 2013; Mendell et al., 2012; Verhaart and Aartsma-Rus, 2012). Several gene therapy strategies are currently under development. They include replacing the mutated gene with a functional candidate gene (gene replacement) or repairing the defective gene by targeted correction and exon skipping (gene repair). Currently, adeno-associated virus (AAV)-mediated gene replacement and antisense oligonucleotide (AON)-mediated exon skipping are at the forefront (see Box 1).

In this Review, we discuss existing DMD animal models and their application in preclinical gene therapy research. We also discuss how to use these models to address the current and emerging challenges in DMD gene therapy.

## Animal modeling of dystrophin deficiency

Both naturally occurring and laboratory-generated animal models are available to study the pathobiology of dystrophin deficiency and to develop innovative therapies for treating DMD. Currently, there are nearly 60 different animal models for DMD, and the list keeps growing (supplementary material Table S1). Non-mammalian (such as *Caenorhabditis elegans*, *Drosophila melanogaster* and zebrafish) and the feline (either hypertrophic or non-hypertrophic) DMD models are rarely used in gene therapy studies (Berger and Currie, 2012; Chamberlain and Benian, 2000; Kunkel et al., 2006; Lloyd and Taylor, 2010; Shelton and Engvall, 2005; Smith, 2011; Winand et al., 1994a), and the newly developed rat and pig DMD models have yet to be used in such research (Hollinger et al., 2014; Klymiuk et al., 2013; Nakamura et al., 2014; Nonneman et al., 2012). As such, we focus this Review on the mouse and dog models (Fig. 2B). We discuss the pros and cons of each system and their use in gene therapy (Table 1).

**Table 1.**
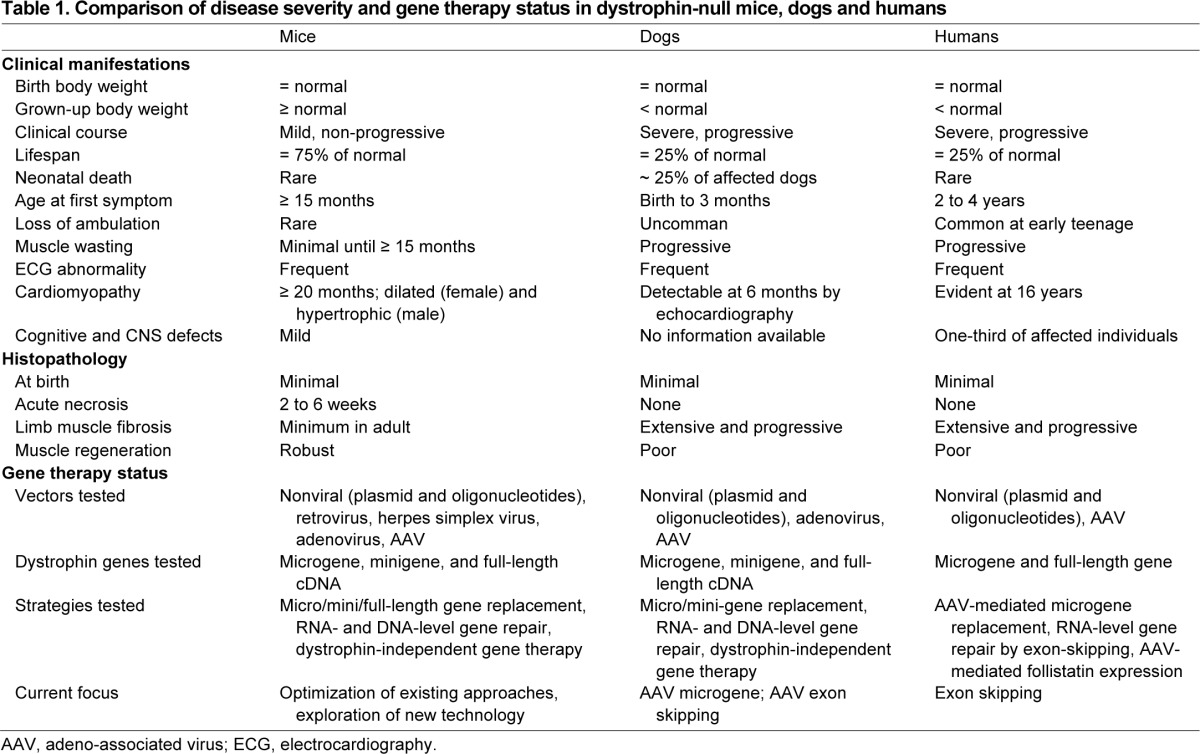
Comparison of disease severity and gene therapy status in dystrophin-null mice, dogs and humans

### Dystrophin-deficient mice

The most widely used animal model for DMD research is the *mdx* mouse. It was discovered in the early 1980s in a colony of C57BL/10ScSn mice due to elevated serum creatine kinase (CK) and histological evidence of myopathy (Bulfield et al., 1984). The mutation in the *mdx* mouse is a nonsense point mutation (C-to-T transition) in exon 23 that aborted full-length dystrophin expression (Fig. 2B) (Sicinski et al., 1989).

**Fig. 3.**
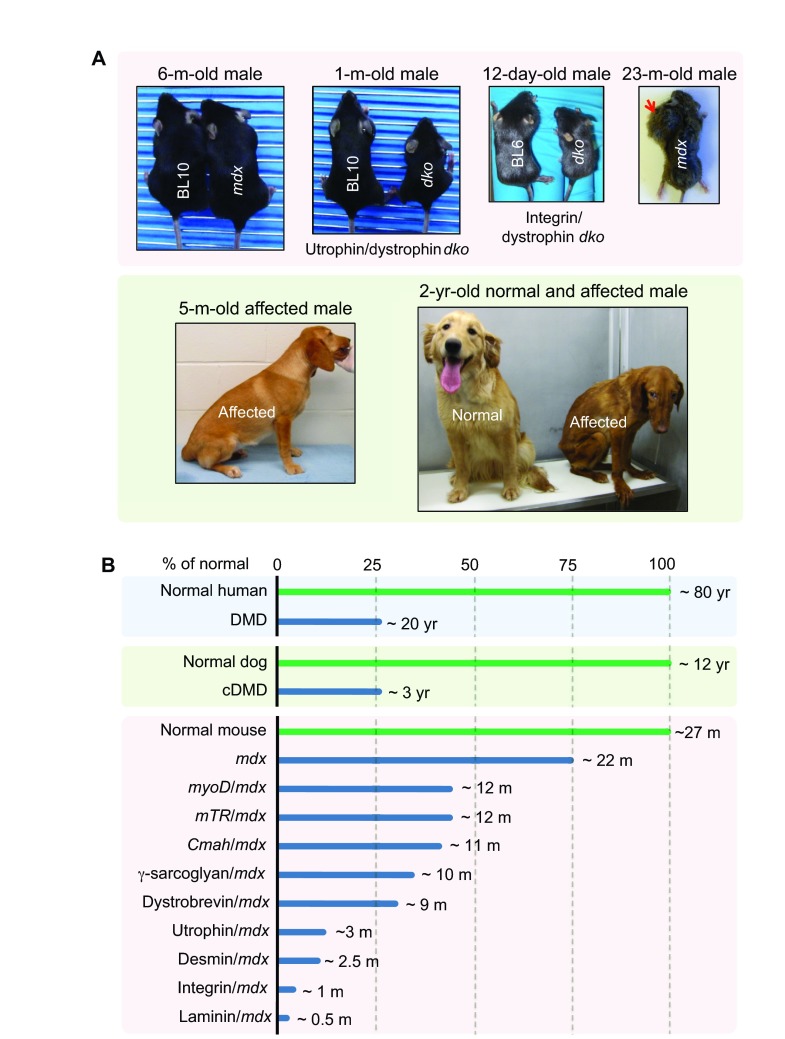
**Representative animal models for DMD.** (A) Representative pictures of selected DMD mouse and dog models. *mdx* mice do not show symptoms (see 6-month-old photo) until very old (see 23-month-old photo). Aged *mdx* mice are also prone to rhabdomyosarcoma (a tumor of muscle origin; red arrow). Utrophin/dystrophin and integrin/dystrophin double-knockout (*dko*) mice are much smaller than the age-matched wild-type (BL10 and BL6) mice. A 5-month-old affected dog shows limb muscle atrophy and is reluctant to exercise. At the age of 2 years old, the affected dog displays severe clinical disease, whereas its normal sibling remains healthy. (B) Lifespan comparison among affected humans, affected dogs and various mouse models.

Despite being deficient for dystrophin, *mdx* mice have minimal clinical symptoms and their lifespan is only reduced by ~25% (Fig. 3; Table 1) (Chamberlain et al., 2007; Li et al., 2009). In contrast, the lifespan of individuals with DMD is reduced by ~75% (Box 2; Fig. 3B). *mdx* skeletal muscle disease has several distinctive phases. In the first 2 weeks, *mdx* muscle is indistinguishable from that of normal mice. Between 3 to 6 weeks, it undergoes startling necrosis. Subsequently, the majority of skeletal muscle enters a relatively stable phase owing to robust regeneration. *mdx* limb muscles often become hypertrophic during this phase. The only exception is the diaphragm, which shows progressive deterioration, as is also seen in affected humans (Box 2) (Stedman et al., 1991). Severe dystrophic phenotypes, such as muscle wasting, scoliosis and heart failure, do not occur until mice are 15 months or older (Bostick et al., 2008b; Bostick et al., 2009; Hakim et al., 2011; Lefaucheur et al., 1995; Lynch et al., 2001; Pastoret and Sebille, 1995). A significant portion of aged *mdx* mice also develops spontaneous sarcoma (Fig. 3A) (Chamberlain et al., 2007; Schmidt et al., 2011; Wang et al., 2014).

**Box 2. Clinical features of DMD**

Large-scale population studies have outlined the natural disease progression in affected humans (Table 1) (Bushby and Connor, 2011; Henricson et al., 2013; Magri et al., 2011; McDonald et al., 2013a; McDonald et al., 2013b; Spurney et al., 2014). The first clinical sign usually appears around age 3. Between ages 5 and 8, symptoms are often stabilized or even slightly improved (known as the ‘honeymoon’ period) in the absence of any treatment (Bushby and Connor, 2011; McDonald et al., 2013a; McDonald et al., 2010). Rapid clinical deterioration starts around 7 to 8 years of age (Mercuri and Muntoni, 2013). Individuals with DMD lose their ambulation at approximately age 10, develop cardiomyopathy at about age 16 and die around age 20 (life expectancy is reduced by ~75%). With the use of steroids, symptom management and multidisciplinary care (especially nocturnal ventilation), the lifespan of an affected individual is now extended to 30 to 40 years of age. In these individuals, cardiac complications (cardiomyopathy and/or cardiac arrhythmia) have emerged as a major source of morbidity and mortality. Despite the overall trend of disease progression throughout life, affected individuals actually show heterogeneity in clinical manifestations. One retrospective study of 75 drug-naïve affected individuals classified DMD into four distinctive groups (infantile, classical, moderate pure motor and severe pure motor) based on the intellectual and motor outcome (Desguerre et al., 2009).

The *mdx* mouse has been crossed to several different genetic backgrounds, including the albino, BALB/c, C3H, C57BL/6, DBA/2 and FVB strains, and several immune-deficient strains. Phenotypic variation has been observed in different backgrounds (supplementary material Table S1). For example, albino-*mdx* mice show more severe neurological dysfunction and higher circulating cytokines (Stenina et al., 2013). BALB/c-*mdx* and C3H-*mdx* mice are less susceptible to sarcoma (Krivov et al., 2009; Schmidt et al., 2011; Stenina et al., 2013). Immune-deficient nude-*mdx* and *scid-mdx* mice show less fibrosis (Farini et al., 2007; Morrison et al., 2000). The DBA/2-*mdx* mice are thought to better represent human disease because they display more fibrosis and less regeneration (Fukada et al., 2010). However, according to The Jackson Laboratory, the DBA/2 strain is a challenging breeder and it also carries mutations in a variety of genes that cause hearing loss and eye abnormalities (http://jaxmice.jax.org/strain/000671.html).

In 1989, four chemical variant (cv) *mdx* strains were published (Chapman et al., 1989). These mice were generated on the C57BL/6 background using the mutagen N-ethyl-N-nitrosourea (ENU) and they are named as *mdx^2cv^*, *mdx^3cv^*, *mdx^4cv^* and *mdx^5cv^*. Each of these strains carries a different point mutation (Fig. 2B; supplementary material Table S1) (Cox et al., 1993b; Im et al., 1996). Although the overall clinical presentation of these mice differs very little from that of *mdx* mice, each line has unique features. Specifically, *mdx^3cv^* mice still express ~5% of a near-full-length dystrophin protein (Cox et al., 1993b; Li et al., 2008). *mdx^5cv^* mice have a more severe skeletal muscle phenotype (Beastrom et al., 2011). Revertant fibers (see Box 1) are rarely seen in *mdx^4cv^* and *mdx^5cv^* mice (Danko et al., 1992; Partridge and Lu, 2008). In addition to these four strains, several new ENU-induced dystrophin-null lines have been recently generated (supplementary material Table S1) (Aigner et al., 2009).

In addition to the above-mentioned strains, several other dystrophin-deficient lines (*Dup2*, *MD-null*, *Dp71-null*, *mdx52* and *mdx βgeo*) have been created using various genetic engineering techniques (see supplementary material Table S1 for details).

Immune-deficient *mdx* strains are dystrophin-null mice that have been crossed to the immune-deficient background. These mice can be used to study cell or gene therapy without the compounding effects of the host immune response. Besides the commonly used nude-*mdx* and *scid*-*mdx* mice (Farini et al., 2007; Morrison et al., 2000), several new lines (*NSG-mdx^4cv^*, *Rag2^−^IL2rb^−^Dmd^−^* and *W41 mdx*) have recently been developed (supplementary material Table S1) (Arpke et al., 2013; Bencze et al., 2012; Vallese et al., 2013; Walsh et al., 2011). These new lines carry additional mutations that further compromise the immune system.

### Mouse models that recapitulate the DMD phenotype

Dystrophin-deficient mice show minimal clinical disease. This could be due to the upregulation of compensatory mechanisms or to a species-specific property of the muscle. Elimination of compensatory mechanisms or humanization of *mdx* mice results in mouse models that recapitulate the dystrophic phenotype of human with DMD. A major function of dystrophin is to strengthen the sarcolemma by cross-linking the ECM with the cytoskeleton. Two other proteins, utrophin and α7β1-integrin fulfil the same function and their expression is upregulated in *mdx* mice. The genetic elimination of utrophin and α7-integrin in *mdx* mice creates utrophin/dystrophin and integrin/dystrophin double-knockout (*dko*) mice, respectively (Deconinck et al., 1997a; Grady et al., 1997; Guo et al., 2006; Rooney et al., 2006). These *dko* mice are significantly smaller than their single-gene null parents and show much more severe muscle disease (similar to or even worse than that of humans with DMD) (Fig. 3A). However, they are difficult to generate and care for, and they often die prematurely (compared with the single knockouts; Fig. 3B). Recent studies suggest that utrophin heterozygous *mdx* mice might represent an intermediate model between the extreme *dko* mice and mildly affected *mdx* mice (Rafael-Fortney et al., 2011; van Putten et al., 2012b; Zhou et al., 2008).

Robust skeletal muscle regeneration also explains the slowly progressive phenotype of *mdx* mice. Two different approaches have been used to reduce muscle regeneration in *mdx* mice. Megeney et al. eliminated MyoD, a master myogenic regulator, from *mdx* mice (Megeney et al., 1996). The resulting MyoD/dystrophin double-mutant mouse shows marked myopathy, dilated cardiomyopathy and premature death (Fig. 3B) (Megeney et al., 1996; Megeney et al., 1999). Compared with normal muscle, the length of telomere is reduced in DMD muscle (Decary et al., 2000). Sacco et al. hypothesized that the long telomere length in mouse myogenic stem cells contributes to the high regenerative capacity of mouse muscle (Mourkioti et al., 2013; Sacco et al., 2010). Telomerase RNA (mTR) is required for the maintenance of the telomere length. To reduce telomere length in dystrophin-null mice, Sacco et al. crossed *mdx^4cv^* mice with *mTR*-null mice. These *mTR/mdx* double-mutant mice show more severe muscle wasting and cardiac defects (Mourkioti et al., 2013; Sacco et al., 2010). Their lifespan is reduced to ~12 months (Fig. 3B).

Other symptomatic *dko* strains (supplementary material Table S1) have also been generated by mutating genes involved in: (1) cytoskeleton-ECM interactions (such as desmin, laminin and like-glycosyltransferase) (Banks et al., 2014; Gawlik et al., 2014; Martins et al., 2013), (2) the DAGC (such as dystrobrevin and δ-sarcoglycan) (Grady et al., 1999; Li et al., 2009), (3) muscle repair (such as dysferlin) (Grady et al., 1999; Han et al., 2011; Hosur et al., 2012; Li et al., 2009) and (4) inflammation and fibrosis [such as interleukin-10, a disintegrin and metalloproteinase protein (ADAM)-8, and plasminogen activator inhibitor-1) (Ardite et al., 2012; Nishimura et al., 2015; Nitahara-Kasahara et al., 2014).

Humanization is another method of increasing *mdx* disease severity. The gene encoding cytidine monophosphate sialic acid hydroxylase (Cmah) is naturally inactivated in humans but not in mice (Varki, 2010). Cmah converts cell-surface sialic acid N-acetylneuraminic acid (Neu5Ac) to N-glycolylneuraminic acid (Neu5Gc). Hence, human cells only have Neu5Ac but no Neu5Gc. Genetic elimination of Cmah humanizes the cell-surface glycan profile in mice (Hedlund et al., 2007). Interestingly, Cmah-deficient *mdx* mice show a more severe phenotype (Fig. 3B). This humanization process renders *Cmah/mdx* mice a better model because they more closely recapitulate human disease (Chandrasekharan et al., 2010).

In summary, the large collection of symptomatic double-mutant mouse lines has greatly expanded the armory of potential mouse models for preclinical studies. Accelerated disease progression in these *dko* mice provides an excellent opportunity not only to obtain results from experimental therapies more rapidly but also to confirm whether a therapy can indeed ameliorate clinically relevant manifestations and increase lifespan. Nevertheless, there are also important limitations. For example, most *dko* mice are difficult to breed and are often not commercially available. Importantly, unlike in humans with DMD, all *dko* mice carry a mutation not only in the dystrophin gene but also in another gene (although because the gene encoding Cmah is inactivated in humans this is not an issue for *Cmah/mdx* mice). This is not the case in affected humans. How this additional mutation influences data interpretation remains incompletely understood.

To study DMD pathogenesis and/or to test effectiveness of certain therapies, *mdx* mice have also been crossed with many other gene-knockout strains that are deficient for additional genes (supplementary material Table S1). It should be noted that these *dko* mice show milder or similar disease phenotypes as that of *mdx* mice (see supplementary material Table S1 for details).

### Dystrophin-deficient dogs

Two major barriers hinder translational DMD gene therapy research. The first is the host cellular and humoral immune responses to the viral capsid and/or therapeutic proteins expressed from the gene therapy vector. The second is the ability to scale-up vector production and to deliver large-scale-produced vector to patients. Unfortunately, mice are not good models for addressing either issue; however, canine models might bridge this gap. Canine X-linked muscular dystrophy has been described in the literature for over 50 years (Duan, 2011; Funkquist et al., 1980; Innes, 1951; Wentink et al., 1972). Confirmed dystrophin deficiency has been reported in ~20 different dog breeds (see supplementary material Table S1 for details). Generally, the clinical phenotype of canine Duchenne muscular dystrophy (cDMD) is considered more severe than that of *mdx* (see below for an in-depth discussion) and, as such, cDMD is regarded as a better model of human DMD.

Dystrophin gene mutations have been mapped in nine cDMD breeds (although only four mutations have been published in peer-reviewed research articles). Point mutations have been found in the Cavalier King Charles spaniel muscular dystrophy dogs (CKCS-MD, intron 50), golden retriever muscular dystrophy dogs (GRMD, intron 6) and Rottweiler muscular dystrophy dogs (exon 52) (Sharp et al., 1992; Walmsley et al., 2010; Winand et al., 1994b). Deletion mutations have been found in three breeds, including a small four-nucleotide deletion in exon 65 (Cocker spaniel), an exon 8–29 deletion in the Tibetan terrier, and a whole-gene deletion in the German shorthaired pointer (Kornegay et al., 2012; Schatzberg et al., 1999). Repetitive element insertions are rarely seen in humans with DMD. However, they have been identified in two dog breeds, including the Pembroke Welsh corgi and the Labrador retriever (Fig. 2B; supplementary material Table S1) (Smith et al., 2007; Smith et al., 2011). A recent genome-walking study suggests that the mutation in Japanese Spitz dystrophic dogs is chromosome inversion (Atencia-Fernandez et al., 2015).

Despite abundant documentation of dystrophin deficiency in dogs, most studies are limited to case reports. Experimental colonies have only been established with a few breeds. The GRMD model is the first and the most widely used cDMD model. It was initially identified by deLahunta and colleagues (Cornell University) and then characterized by Drs Cooper and Kornegay at Cornell University and North Carolina State University, USA, respectively (Cooper et al., 1988b; Hoffman and Gorospe, 1991; Kornegay et al., 1988; Valentine et al., 1986). Subsequent molecular, histological and clinical studies validated GRMD dogs as an authentic model for human DMD (Cooper et al., 1990; Cooper et al., 1988a; Cozzi et al., 2001; Kornegay et al., 1988; Lanfossi et al., 1999; McCully et al., 1991; Moise et al., 1991; Nguyen et al., 2002; Sharp et al., 1992; Valentine et al., 1989a; Valentine et al., 1990a; Valentine et al., 1991; Valentine and Cooper, 1991; Valentine et al., 1990b; Valentine et al., 1986; Valentine et al., 1988; Valentine et al., 1989b; Valentine et al., 1989c; Valentine et al., 1989d; Valentine et al., 1992). Currently, GRMD dogs are maintained in several colonies throughout the USA (including the University of Missouri and Texas A&M University, among others), and in France, Brazil and Australia. The GRMD mutation has also been crossed to the Beagle background and a colony is now maintained in Japan; these dogs are called canine X-linked muscular dystrophy in Japan or CXMD_J_ (Shimatsu et al., 2003; Valentine et al., 1988). Recently, we and others have created hybrid strains that are on mixed genetic backgrounds and/or contain mutations of different breeds (Cotten et al., 2013; Fine et al., 2011; Miyazato et al., 2011; Shin et al., 2013a; Yang et al., 2012). Besides GRMD-based colonies, research colonies have also been generated from affected Pembroke Welsh corgis and Labrador retrievers (Auburn University and University of Missouri), and CKCS-MD (Royal Veterinary College, UK) (Smith et al., 2007; Smith et al., 2011; Walmsley et al., 2010). The CKCS-MD model is especially interesting because the mutation in this breed corresponds to a major deletion hot spot (exons 45–53) in humans with DMD (Aartsma-Rus et al., 2006; Flanigan et al., 2009b; Tuffery-Giraud et al., 2009).

Affected dogs share a remarkably similar clinical course to that of DMD boys (Box 2; Fig. 3; Table 1) (Shimatsu et al., 2005; Smith et al., 2011; Valentine et al., 1988). Limb weakness and exercise intolerance start around 2 to 3 months of age (analogous to ~3 years of age in humans) (Valentine et al., 1988). Muscle atrophy, joint contracture, hypersalivation, dysphagia, abnormal gait and signs of cardiac involvement become apparent at ~6 months (Fig. 3A; Table 1) (Fan et al., 2014; Fine et al., 2011; Valentine et al., 1988; Valentine et al., 1989c; Yugeta et al., 2006). At around 6 to 10 months, disease progression enters a relatively stable ‘honeymoon’ period (Fan et al., 2014; Shimatsu et al., 2005; Valentine et al., 1988). Death often occurs around 3 years of age (a ~75% reduction of the lifespan) (Fig. 3B). Humans with DMD show heterogeneity in their clinical manifestation (Box 2) (Ashwath et al., 2014; Desguerre et al., 2009; Sifringer et al., 2004). cDMD dogs also show variation in their symptoms. In extreme cases, affected subjects are essentially asymptomatic despite the lack of dystrophin in their muscles (Ambrósio et al., 2008; Dubowitz, 2006; Hattori et al., 1999; Wakefield et al., 2009; Zatz et al., 2014; Zucconi et al., 2010).

Besides clinical resemblance, cDMD dogs also have histological lesions similar to affected humans. For example, limb muscle fibrosis is a salient disease feature in humans with DMD and in affected dogs but not in *mdx* mice (C.H.H. and D.D., unpublished observations). Vigorous regeneration in mouse muscle contributes substantially to the mild phenotype of *mdx* mice. This regeneration is evident by high proportions of centrally nucleated myofibers in *mdx* mice. Similar to humans with DMD, cDMD dogs have much fewer myofibers containing central nucleation (Cozzi et al., 2001; Shin et al., 2013b; Smith et al., 2011; Yang et al., 2012).

It should be noted that the clinical presentation of cDMD dogs is not identical to that of humans with DMD (Table 1). About 20–30% of cDMD puppies die within 2 weeks of birth likely due to diaphragm failure (Ambrósio et al., 2008; Nakamura et al., 2013; Shimatsu et al., 2005; Valentine et al., 1988). However, this neonatal death is not seen in newborn DMD boys. Growth retardation is another canine-specific symptom (West et al., 2013). Body weight at birth is similar between normal and affected cDMD puppies (Smith et al., 2011). However, at 1 and 6 months of age, the body weight of affected puppies reaches only ~80% and ~60% of normal, respectively (C.H.H. and D.D., unpublished observation from *n*>50 dogs). Finally, untreated humans with DMD usually lose ambulation during the early teenage years. However, complete loss of ambulation is not a clinical feature in young cDMD dogs (Duan et al., 2015; Valentine et al., 1988).

Overall, cDMD dogs share many features with that of humans with DMD. These features make cDMD dogs an excellent model to conduct preclinical gene therapy studies (Duan, 2011; Duan, 2015). Nevertheless, *mdx* mice remain the most commonly used model in DMD gene therapy studies owing to the low cost and easy access. Any discussion of DMD models in gene therapy that lacked mention of *mdx* mice would not be complete.

## Establishing the foundations of gene therapy: transgenic *mdx* mice

The successful development of a gene therapy requires research to identify the therapeutic candidate gene, the level of expression needed to produce a therapeutic effect and the tissue that should be targeted (Chamberlain, 2002; Duan, 2006). As we discuss in this section, for DMD gene therapy research, these fundamental questions have been addressed using transgenic *mdx* mice.

### Therapeutic potential of truncated dystrophin genes

#### Naturally occurring small dystrophin isoforms

**Fig. 4.**
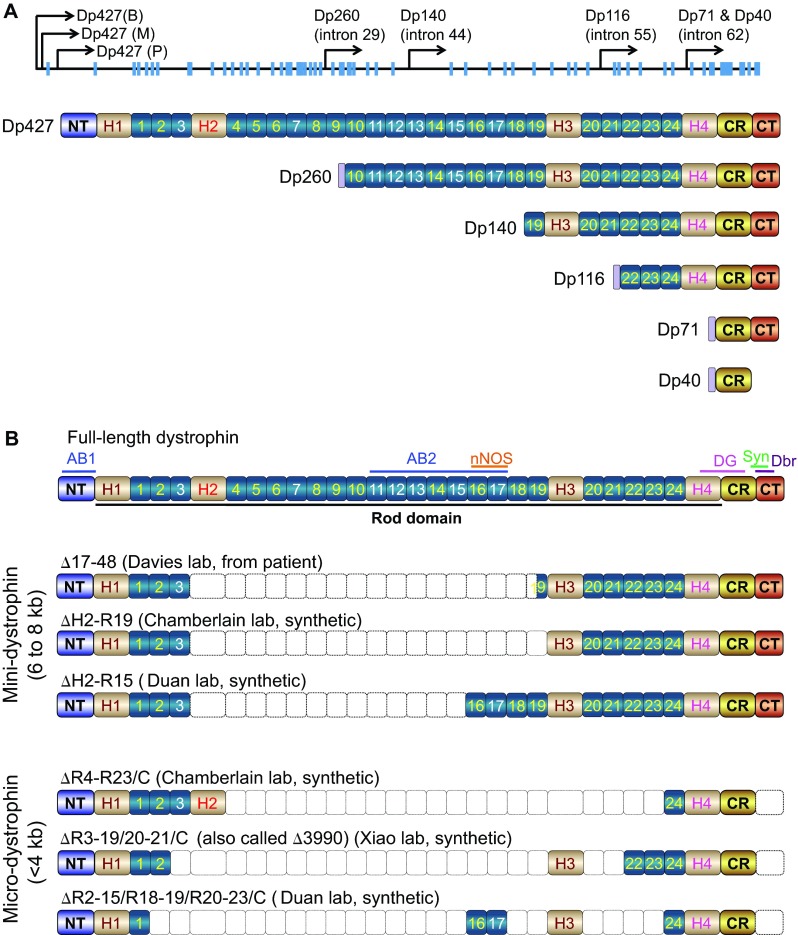
**Structure of abbreviated dystrophins.** (A) Naturally occurring dystrophin isoforms. In the topmost schematic, blue boxes denote exons. The full-length dystrophin (Dp427) transcripts have three isoforms, including brain Dp427 (B), muscle Dp427 (M) and Purkinje cell Dp427 (P). Smaller dystrophin isoforms are produced from promoters located in different introns (intron positions are marked for each isoform). Dp260 is expressed in the retina, Dp140 in the brain and kidney, Dp116 in Schwann cells, and Dp71 and Dp40 are expressed from the same promoter except Dp71 is ubiquitously expressed whereas Dp40 only exists in the brain. Except for Dp140, all other dystrophin isoforms have unique N-terminal sequences not present in the full-length protein. (B) Structure of representative mini- and micro-dystrophins. The full-length dystrophin protein is shown uppermost, and features the same terminology as that used in Fig. 1.

The enormous size of the full-length dystrophin gene poses one of the biggest challenges for gene therapy because it exceeds the packaging limit of most viral vectors. For this reason, identifying a smaller but functional gene has been an ongoing goal in the development of a dystrophin gene-replacement therapy. Early studies showed that, besides the 427-kDa full-length protein, the dystrophin gene also encodes a number of smaller N-terminal-truncated non-muscle isoforms (Ahn and Kunkel, 1993; Blake et al., 2002; Ervasti, 2007). These include Dp260, Dp140, Dp116, Dp71 and Dp40 (numbers refer to the molecular weight) (Fig. 4A). With the exception of Dp40 (Fujimoto et al., 2014), they all contain the CT and CR domains but are missing the NT actin-binding domain. To determine whether these miniature isoforms are therapeutically relevant, the Chamberlain lab, as well as others, made transgenic *mdx* mice for Dp260, Dp116 and Dp71 (see supplementary material Table S1 for details) (Cox et al., 1994; Gaedigk et al., 2006; Greenberg et al., 1994; Judge et al., 2011; Judge et al., 2006; Warner et al., 2002).

Dp71 is the most abundant non-muscle dystrophin isoform. It contains only the CR and CT domains (Fig. 4A). Because the CT domain carries the binding sites for syntrophin and dystrobrevin, it was initially thought that Dp71 might restore some of the signaling functions of dystrophin. Surprisingly, however, transgenic overexpression of Dp71 results in more severe muscle disease in *mdx* mice (Cox et al., 1994; Greenberg et al., 1994) and myopathy in normal mice (Leibovitz et al., 2002). The WW domain of hinge 4 (H4; see Fig. 1A and Box 1), which is partially truncated in Dp71, participates in dystrophin-dystroglycan interaction (Huang et al., 2000). To fully appreciate the contribution of dystroglycan binding and dystrophin signaling in DMD pathogenesis, Judge et al. generated Dp116 transgenic *mdx* mice. Dp116 is a Schwann-cell-specific dystrophin isoform. It contains the last three spectrin-like repeats, H4, and the CR and CT domains (Fig. 4A). Dp116 expression does not improve muscle disease in *mdx^4cv^* mice nor does it reduce the histopathology in utrophin/dystrophin *dko* mice (Judge et al., 2011; Judge et al., 2006). Interestingly, the lifespan of utrophin/dystrophin *dko* mice was significantly increased by transgenic *Dp116* expression (Judge et al., 2011).

Two independent strains of Dp260 transgenic mice have been studied (Gaedigk et al., 2006; Warner et al., 2002). Although Dp260 (also known as the retinal isoform of dystrophin) does not carry the NT domain, it contains the ABD2 domain (Fig. 2B; Fig. 4A). Its overexpression significantly reduces the dystrophic phenotype of *mdx* and utrophin/dystrophin *dko* mice but does not completely prevent muscle degeneration, inflammation and fibrosis (Gaedigk et al., 2006; Warner et al., 2002). In summary, transgenic analyses of naturally occurring dystrophin isoforms suggest that the N-terminal domain is required for maximum muscle protection and that a complete dystroglycan-binding domain (including the WW domain in H4 and the CR domain) is important.

#### Synthetic mini- and micro-dystrophin genes

An alternative approach to developing a smaller but functional dystrophin gene is through genetic engineering. To achieve this, one needs to know which regions of the dystrophin gene are dispensable for its normal functions. The first clue about this came from a mildly affected individual, who was ambulant at age 61 (England et al., 1990). This person carries a large in-frame deletion (Δ17–48) in the rod domain, which eliminates 46% of the coding sequence. Transgenic expression of the Δ17–48 minigene in *mdx* mice significantly reduced skeletal muscle pathology and increased specific muscle force (Phelps et al., 1995; Wells et al., 1995). Subsequent optimization by removing residue repeat 19 in the Δ17–48 minigene resulted in a more protective ΔH2-R19 minigene (Fig. 4B) (Harper et al., 2002).

An important function of dystrophin is to recruit nNOS to the sarcolemma. Failure to do so results in functional ischemia and aggravates muscle disease (Thomas, 2013). We recently identified the dystrophin nNOS-binding site at R16/17 of the rod domain **(**Fig. 1; Fig. 2B) (Lai et al., 2009; Lai et al., 2013). Inclusion of this binding site in synthetic dystrophins (Fig. 4B) significantly enhances muscle protection and exercise capacity (Lai et al., 2009; Zhang et al., 2013).

The mini-dystrophin gene is ~6 to 8 kb. One drawback is that it cannot fit into the 5-kb packaging limit of AAV, the most efficient muscle gene-transfer vector. A pivotal transgenic study from the Chamberlain lab opened the door to further reducing the size of the dystrophin gene by deleting the entire CT domain (Crawford et al., 2000). Specifically, Chamberlain and colleagues showed that a C-terminal-truncated dystrophin gene successfully restored syntrophin and dystrobrevin to the sarcolemma and completely protected young adult *mdx* mice (Crawford et al., 2000). Consistent with this transgenic study, a subset of affected individuals who have partial or complete CT-domain deletion also show mild disease (Aartsma-Rus et al., 2006; McCabe et al., 1989; Patria et al., 1996; Tuffery-Giraud et al., 2009). Collectively, the existing data suggest that the majority of the rod domain (except for R16/17) and the entire C-terminal domain are not essential for dystrophin function. Based on this understanding, several versions of highly abbreviated synthetic micro-dystrophin genes (<4 kb) have been engineered (Fig. 4B) (Harper et al., 2002; Lai et al., 2009; Wang et al., 2000). These microgenes greatly prevent muscle damage in transgenic *mdx* mice (Hakim and Duan, 2013; Harper et al., 2002; Li et al., 2011).

### Level of expression

Two essential questions in DMD gene therapy are: (1) how much dystrophin is too much, and (2) how much dystrophin is enough to ameliorate disease? In transgenic *mdx* mice, Chamberlain and colleagues found that 50-fold overexpression of full-length dystrophin was not toxic to skeletal muscle, thus providing a high safety margin (Cox et al., 1993a). Studies in transgenic *mdx* mice have also revealed the threshold for histological and physiological protection (Phelps et al., 1995; Wells et al., 1995). Dystrophin expression at ~20% of the wild-type level significantly mitigated muscle pathology and enhanced muscle contractility (Phelps et al., 1995; Wells et al., 1995). *mdx^3cv^* mice express ~5% of a near-full-length dystrophin protein and *mdx-Xist^Δhs^* mice express variable low levels of dystrophin (supplementary material Table S1) (Cox et al., 1993b; van Putten et al., 2012a). Recent studies in *mdx^3cv^* and *mdx-Xist^Δhs^* mice suggest that dystrophin expression at a 5% level still preserves some muscle function in *mdx* mice and extends the lifespan of utrophin/dystrophin *dko* mice (Li et al., 2008; Li et al., 2010; van Putten et al., 2012a; van Putten et al., 2013). A clear correlation between the dystrophin level and clinical manifestation has also been noticed in humans with DMD (Nicholson et al., 1993a; Nicholson et al., 1993b). Affected individuals with ≥20% wild-type dystrophin protein expression are often ambulant beyond age 20 (Bulman et al., 1991; Byers et al., 1992; Hoffman et al., 1989). An affected individual with 30% dystrophin protein expression, measured through western blot, was even free of skeletal muscle disease at age 23 (Neri et al., 2007). Where gene therapy is concerned, there is no doubt that restoring ≥20% protein expression will be needed to achieve clinically meaningful improvement. Nonetheless, mouse data suggest that even a low level of expression (~5%) might still be beneficial.

### Target tissue: skeletal muscle versus heart

Humans with DMD suffer from both skeletal muscle disease and cardiomyopathy. It thus seems obvious that both skeletal and heart muscle should be treated. However, many existing gene therapy approaches (such as some AONs used for exon skipping and AAV serotype-9-mediated systemic gene transfer in newborn dogs) cannot efficiently reach the heart (Alter et al., 2006; Hakim et al., 2014; Yokota et al., 2009; Yue et al., 2008). Will skeletal-muscle-centered therapy benefit individuals with DMD? An early study in young (4- to 5-month-old) transgenic *mdx^4cv^* mice suggests that targeted repair of skeletal muscle accelerates heart disease (Townsend et al., 2008). However, the interpretation of the heart function data in this study has been questioned (Wasala et al., 2013). Using a different approach, Crisp et al. reached a completely opposite conclusion in adult (6- to 9-month-old) *mdx* mice and neonatal (10-day-old) utrophin/dystrophin *dko* mice (Crisp et al., 2011). They concluded that skeletal muscle rescue can prevent cardiomyopathy (Crisp et al., 2011). Because *mdx* mice do not develop clinically evident cardiomyopathy until they are 21-months old (Bostick et al., 2008b; Bostick et al., 2009), we recently re-evaluated this issue in a similar transgenic strain used by Townsend et al. (Townsend et al., 2008; Wasala et al., 2013). Surprisingly, skeletal-muscle-rescued *mdx* mice showed the identical heart disease as that of non-transgenic *mdx* mice at the age of 23 months (Wasala et al., 2013). In summary, skeletal muscle rescue might neither aggravate nor completely alleviate cardiomyopathy. As such, we believe that gene therapy should treat both skeletal and cardiac muscles.

## Gene replacement therapy

A straightforward approach to treating DMD is to add back a functional dystrophin gene. This can be achieved using a variety of gene-transfer vectors, including nonviral, retroviral, adenoviral, herpes simplex viral and AAV vectors. The candidate gene can be the full-length cDNA or an abbreviated synthetic gene.

### Replacement with the full-length dystrophin coding sequence

Several strategies have been explored to deliver the 14-kb, full-length dystrophin cDNA. Direct plasmid injection was tested in *mdx* mice soon after the discovery of the dystrophin gene (Acsadi et al., 1991). A number of different nonviral delivery approaches have since been evaluated in *mdx* mice. These include the use of liposomes, microspheres, electroporation and hydrodynamic intravascular delivery (see Box 1). Direct plasmid injection has also been tested in the GRMD model and in a Phase 1 human trial (Braun, 2004; Duan, 2008). However, poor transduction and transient expression have limited further development of these plasmid-based therapeutic strategies.

The gutted adenoviral vector does not carry any viral genes and can package a 35-kb genome. It has been used to express the full-length dystrophin cDNA (Haecker et al., 1996; Kochanek et al., 1996; Kumar-Singh and Chamberlain, 1996). Tests conducted in *mdx* and utrophin/dystrophin *dko* mice have yielded promising results (Clemens et al., 1996; DelloRusso et al., 2002; Ishizaki et al., 2011; Kawano et al., 2008). The current challenges are the host immune response to the adenoviral capsid and the contaminating wild-type adenovirus. Herpes simplex virus also has an extremely large capacity (~150 kb) and has been used to package the full-length dystrophin cDNA (Akkaraju et al., 1999; Liu et al., 2006). However, there have been very few animal studies performed with it due to the toxicity of the virus.

Recently, tri-AAV vectors were used to deliver the full-length dystrophin cDNA (see Box 1) (Koo et al., 2014; Lostal et al., 2014). In this system, the full-length cDNA expression cassette is split into three fragments and separately packaged in an AAV vector. Coinfection with all three AAV vectors results in the production of a full-length dystrophin protein. This approach has been tested in *mdx* and *mdx^4cv^* mice by direct muscle injection. The therapeutic benefits of this system await substantial improvement in transduction efficiency.

### Replacement with small synthetic dystrophin genes

The 6- to 8-kb minigenes discussed earlier in the Review have been tested with plasmid, retrovirus, adenovirus and AAV. Retroviral delivery is very inefficient because the virus does not transduce post-mitotic muscle cells (Dunckley et al., 1993). The first-generation E1-deleted adenovirus was used to deliver the Δ17–48 minigene to *mdx* mice and GRMD dogs (Howell et al., 1998; Ragot et al., 1993). Although this vector is more efficient than a retroviral vector, it induces a strong cellular immune response in *mdx* mice (Howell et al., 1998; Ragot et al., 1993). The Chamberlain and Duan labs have tested dual-AAV-vector-mediated mini-dystrophin therapy in *mdx* mice using local and systemic gene transfer (see Box 1) (Ghosh et al., 2008; Lai et al., 2005; Odom et al., 2011; Zhang and Duan, 2012; Zhang et al., 2013). In the dual AAV vector system, mini-dystrophin expression is achieved with a pair of AAV vectors, each carrying half of the minigene. These studies have shown a significant improvement of histology and function in treated *mdx* mice. Noticeably, the use of the R16/17-containing mini-dystrophin dual AAV vectors has successfully restored sarcolemmal nNOS expression and ameliorated functional ischemia (Zhang and Duan, 2012; Zhang et al., 2013).

AAV-mediated, micro-dystrophin gene therapy is currently at the cutting edge of DMD gene-replacement therapy. Local injection studies performed in the Chamberlain, Dickson, Duan, Takeda and Xiao laboratories suggest that a rationally designed dystrophin microgene can protect limb muscles and the heart in *mdx* mice despite the absence of ~70% of the coding sequence (Harper et al., 2002; Wang et al., 2000; Yoshimura et al., 2004; Yue et al., 2003). Using the newly developed AAV serotype-6 and -8 vectors (Gao et al., 2002; Rutledge et al., 1998), the Chamberlain and Xiao labs achieved widespread whole-body muscle gene transfer in the rodent models of muscular dystrophies (Gregorevic et al., 2004; Wang et al., 2005). Later, it was found that AAV serotype-9 can also provide efficient systemic muscle delivery (Bostick et al., 2007; Pacak et al., 2006). More recent studies suggest that AAV-8 and AAV-9 can also produce robust body-wide muscle gene transfer in neonatal dogs (Hakim et al., 2014; Kornegay et al., 2010; Pan et al., 2013; Yue et al., 2008).

The first systemic gene therapy test was performed in *mdx* mice by Gregorevic et al. (Gregorevic et al., 2004) and subsequently in utrophin/dystrophin and myoD/dystrophin *dko* mice (Gregorevic et al., 2006; Lai et al., 2009). In these studies, micro-dystrophin gene therapy significantly ameliorated the histological and physiological signs of muscular dystrophy, reduced CK levels and extended lifespan. To further improve therapeutic efficacy, several labs made additional changes to the existing micro-dystrophin constructs. Dickson and colleagues found that codon-optimization and inclusion of the syntrophin/dystrobrevin-binding site resulted in better rescue (Foster et al., 2008; Koo et al., 2011a). The Chamberlain lab found that the rigid poly-proline site in hinge 2 compromised micro-dystrophin function (Banks et al., 2010). Our studies have suggested that R16/17 should be incorporated in the microgene design to normalize nNOS expression (Harper, 2013; Lai et al., 2009; Lai et al., 2013; Li et al., 2011; Shin et al., 2013b).

In an effort to translate AAV microgene therapy to large mammals, several groups have extended research into cDMD models (Koo et al., 2011b; Kornegay et al., 2010; Shin et al., 2012a; Shin et al., 2012b; Wang et al., 2007). These studies uncovered two important issues that were not encountered during mouse studies. First, intramuscular injection results in a strong cellular immune response (Ohshima et al., 2009; Wang et al., 2007; Yuasa et al., 2007; Yue et al., 2008). As a result, transient immune suppression is necessary for persistent transduction in dog muscle (Shin et al., 2012b; Wang et al., 2007). Second, a microgene that reduces muscle disease in mice might not work effectively in dogs (Kornegay et al., 2010; Sampaolesi et al., 2006). Specifically, the ΔR4-23/C dystrophin microgene did not improve muscle histology when tested in a cell therapy study (Sampaolesi et al., 2006). Newborn GRMD dogs developed more severe disease after treatment with the ΔR3-19/20–21/C (also called Δ3990) microgene (Kornegay et al., 2010). Currently, convincing physiological improvement has only been demonstrated in the ΔR2-15/R18-19/R20-23/C microgene-treated dogs (Shin et al., 2013b).

## Gene repair therapy

Therapeutic approaches that aim to repair or correct a DMD gene mutation have been conducted at both the RNA and DNA level using oligonucleotides or engineered endonucleases (Aartsma-Rus, 2012; Bertoni, 2014). Although AON-mediated exon skipping has already reached Phase 3 human trials, endonuclease-based gene repair has just begun to emerge (Koo and Wood, 2013; Lu et al., 2011).

### Repairing the dystrophin transcript

Therapeutic RNA targeting using exon skipping is by far the most advanced DMD gene therapy technology developed to date. In exon skipping, AONs are used to modulate the splicing of the RNA transcript such that one or several exons are excluded. As a result, an out-of-frame mRNA is converted into an in-frame transcript or an exon that contains a premature stop codon is removed from the transcript (Spitali and Aartsma-Rus, 2012). An internally deleted but partially functional dystrophin produced from exon skipping is expected to convert severe DMD to the milder Becker phenotype. This approach represents an excellent example of how a rationally designed strategy can rapidly move from bench to bedside.

**Fig. 5.**
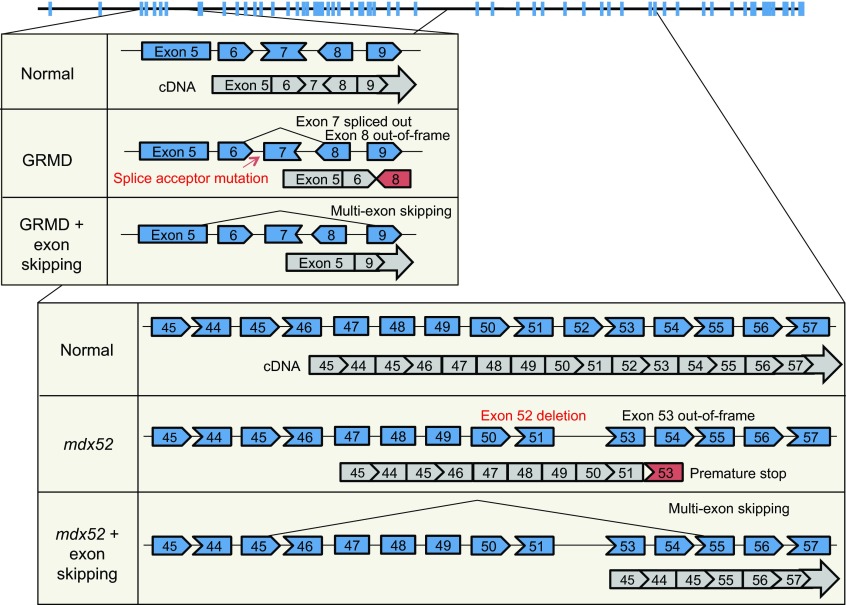
**Multiple-exon skipping.** The uppermost diagram is the intron/exon structure of the dystrophin gene. Blue boxes denote exons. The top box shows the golden retriever muscular dystrophy dog (GRMD) mutation and exon skipping for GRMD. A point mutation in intron 6 alters normal splicing, and the resulting transcript (gray) is out-of-frame. Skipping exons 6, 7 and 8 yields an in-frame transcript. The bottom box shows the *mdx52* mutation and exon skipping in *mdx52*. Deletion of exon 52 disrupts the reading frame and results in a premature stop. Removing exons 45 to 55 from the mutated transcript generates an in-frame transcript.

The initial proof-of-principle study for exon skipping was conducted in cultured *mdx* mouse muscle cells (Dunckley et al., 1998). Subsequent *in vivo* tests in *mdx* mice showed that this approach produced a highly efficient restoration of dystrophin expression and improved muscle function, following local or systemic injection (Alter et al., 2006; Gebski et al., 2003; Lu et al., 2003; Lu et al., 2005; Mann et al., 2001). Similarly, exon skipping (Fig. 5) has been achieved in cultured cDMD muscle cells and in CXMD_J_ dogs by local and systemic delivery (McClorey et al., 2006; Walmsley et al., 2010; Yokota et al., 2009). Several clinical trials have been initiated based on the results of animal studies (Koo and Wood, 2013; Opar, 2012). Data from the Phase 1 and 2 trials are highly promising (Cirak et al., 2011; Goemans et al., 2011; Kinali et al., 2009; Mendell et al., 2013; van Deutekom et al., 2007). However, the expected efficacy remains to be confirmed in a Phase 3 study (Hoffman and McNally, 2014; Wood, 2013).

Early exon-skipping studies used AONs based on 2′-O-methylated phosphorothioate (2OMe-PS) or phosphorodiamidate morpholino oligomers (PMOs) (Box 1). An important limitation of these AONs is that they cannot reach the heart. To overcome this hurdle, a variety of conjugated PMOs have been developed (Aoki et al., 2012; Jearawiriyapaisarn et al., 2008; Wu et al., 2009; Wu et al., 2008; Yin et al., 2008; Yin et al., 2011). In these PMOs, oligonucleotides are covalently linked to a cell-penetrating peptide or an octa-guanidine dendrimer, which can enhance cell penetration (the octa-guanidine-modified PMO is called vivo-morpholino; see Box 1). Systemic delivery of conjugated AONs in *mdx* mice produced robust exon skipping in the heart and the restoration of cardiac function (Wu et al., 2008; Wu et al., 2011). Another drawback of AON therapy is the rapid turnover of the therapeutic oligonucleotides. To solve this problem, investigators have begun to use the AAV vector to achieve persistent AON delivery *in vivo* in *mdx* mice (Denti et al., 2006; Goyenvalle et al., 2004). Recently, AAV-based exon skipping has been shown to significantly improve the dystrophic phenotype in utrophin/dystrophin *dko* mice and in GRMD dogs (Barbash et al., 2013; Bish et al., 2012; Goyenvalle et al., 2012; Le Guiner et al., 2014; Vulin et al., 2012).

*mdx* mice and GRMD dogs carry point mutations in the dystrophin gene. However, ~ 60% of DMD is due to deletions in exons 45–53 or duplications in exon 2 (Flanigan et al., 2009b). *mdx52* and *dup2* mice carry mutations that resemble the deletions and duplications in affected humans, respectively. Hence, they are excellent models for preclinical testing. Aoki et al. delivered a cocktail of ten vivo-morpholino AONs to *mdx52* mice and achieved efficient multiple-exon skipping (exons 45–55) (Fig. 5) (Aoki et al., 2012). The resulting Δ45–55 dystrophin transcript is highly protective and significantly improves muscle strength and histology without causing any toxicity (Aoki et al., 2012). The duplication of exon 2 is a more challenging error to correct because a complete skipping of exon 2 leads to an out-of-frame transcript. Wein et al. recently tested exon 2 skipping in the *dup2* model using an AAV-based exon-skipping system (Wein et al., 2014). The treatment generated a Δ2 transcript with a premature stop codon in exon 3. Surprisingly, however, the dystrophic phenotype was significantly ameliorated. Further investigation suggests that the removal of exon 2 activates a downstream internal ribosome entry site in exon 5. Translation from this site yields a highly functional protein (Wein et al., 2014). The results of the Aoki et al. and Wein et al. studies are especially appealing because humans who carry similar transcripts are often asymptomatic (Ferreiro et al., 2009; Flanigan et al., 2009a; Nakamura et al., 2008). Therapies based on the same principle might therefore yield dramatic clinical improvement in boys with DMD.

### Repair at the DNA level

Compared to exon skipping, approaches to correct the mutated dystrophin gene are less developed (Bertoni, 2014). Initial DNA-repair strategies used oligonucleotides that are homologous to the target DNA. This approach has resulted in gene correction in *mdx* and *mdx^5cv^* mice, and in one GRMD dog, but the efficiency was too low for clinical application (Bartlett et al., 2000; Kayali et al., 2010; Rando et al., 2000). Nuclease-based gene editing is a powerful technology to correct DNA defects (Box 1). Briefly, a nuclease is used as a pair of molecular scissors to cut DNA at the target site. When a double-strand DNA break is repaired by cellular mechanisms, insertions and/or deletions are introduced at the break point. Some of these modifications yield the wild-type sequence, hence gene correction. Four families of engineered nucleases have been recently developed, including meganuclease, zinc-finger nuclease, TALEN (transcription activator-like effector nuclease) and the CRISPR/Cas (clustered regularly interspaced short palindromic repeat/CRISPR-associated nuclease/helicase) system. These have all been explored for use in DMD therapy; however, the majority of the studies are currently limited to cultured cells (Chapdelaine et al., 2010; Long et al., 2014; Ousterout et al., 2014; Ousterout et al., 2013; Rousseau et al., 2011). Future studies are needed to validate these highly promising gene-editing strategies in animal models of DMD.

## Gene therapy for cardiomyopathy and neuronal defects

Cardiomyopathy and neuronal defects are two other prominent clinical features of DMD. Gene therapy for the heart and central nervous system (CNS) requires special consideration (Anderson et al., 2002; Duan, 2006; Lai and Duan, 2012; Nardes et al., 2012; Ricotti et al., 2011; Shin et al., 2010; Snow et al., 2013) because these organs differ from skeletal muscle in their anatomy and physiology. Importantly, dystrophin deficiency produces a unique disease profile in the heart and CNS.

### Duchenne cardiomyopathy gene therapy

The characteristic cardiac manifestation of DMD is dilated cardiomyopathy (Duan, 2006; Finsterer and Cripe, 2014). Heart damage is also a prominent phenotype in various strains of *dko* mice, including the utrophin/dystrophin *dko*, α7-integrin/dystrophin *dko*, myoD/dystrophin *dko* and mTR/dystrophin *dko* mice (Grady et al., 1997; Guo et al., 2006; Megeney et al., 1999; Mourkioti et al., 2013) (supplementary material Table S1). However, aged female *mdx* mice are by far the best mouse models for studying Duchenne dilated cardiomyopathy because they are genetically and phenotypically identical to affected humans (Bostick et al., 2010; Bostick et al., 2008b).

Most Duchenne cardiomyopathy gene therapy studies have been conducted in the *mdx* model. Using dystrophin heterozygous mice, Duan and colleagues demonstrated that dystrophin expression in 50% of cardiomyocytes was sufficient to mitigate heart injury in *mdx* mice (Bostick et al., 2008b; Yue et al., 2004). The first cardiac gene therapy study was performed in neonatal *mdx* mice using an AAV-5 ΔR4-23/ΔC microgene vector in our laboratory (Fig. 4B). This micro-dystrophin gene therapy restores the DAGC and increases the strength of the cardiomyocyte membrane (Yue et al., 2003). Subsequent studies using the same microgene normalized the electrocardiography (ECG) defects and improved cardiac hemodynamics in young and adult *mdx* mice (Bostick et al., 2008a; Schinkel et al., 2012; Shin et al., 2011b; Townsend et al., 2007). To further explore the therapeutic potential, Bostick et al. treated aged female *mdx* mice with an AAV-9 ΔR4-23/ΔC microgene vector (Bostick et al., 2011; Bostick et al., 2012). They achieved efficient whole-heart gene transfer despite the presence of extensive myocardial fibrosis. In near-terminal-age mice (16- to 20 months old), fibrosis was significantly reduced and hemodynamic performance significantly enhanced (Bostick et al., 2011). However, such improvements were not observed in terminal-age mice (>21 months old) (Bostick et al., 2012). The cardiac protection of the mini-dystrophin gene has only been examined using the 6-kb ΔH2-R19 minigene in transgenic *mdx* mice (Fig. 4B) (Bostick et al., 2009). This minigene completely normalizes skeletal muscle force in transgenic *mdx* mice (Harper et al., 2002). However, it does not lead to a full recovery of heart function (Bostick et al., 2009).

Exon skipping has also been explored for treating *mdx* heart disease. The original 2OMe-PS and PMO AONs cannot reach the heart (Alter et al., 2006). However, conjugated PMOs developed in the Lu and Wood labs have significantly increased cardiac exon skipping and heart contractility in *mdx* mice (Wu et al., 2009; Wu et al., 2008; Wu et al., 2011; Yin et al., 2008; Yin et al., 2011). Recently, two groups tested AAV-based exon skipping in GRMD dogs. Sweeney and colleagues delivered the vector to the heart via fluoroscopy-guided trans-endocardial injection (Bish et al., 2012). This treatment restored dystrophin expression in the heart, reduced fibrosis and improved left ventricular function (Bish et al., 2012). Using X-ray-fused magnetic resonance, Barbash et al. have further improved the transendocardial gene-delivery method and achieved dystrophin expression in the GRMD heart (Barbash et al., 2013).

### Correcting neuronal defects with gene therapy

About one-third of individuals with DMD display cognitive deficiency and other CNS symptoms (Anderson et al., 2002; D’Angelo and Bresolin, 2006; Nardes et al., 2012; Ricotti et al., 2011; Snow et al., 2013). Although all dystrophin isoforms have been detected in the nervous system (Lidov, 1996; Tozawa et al., 2012), only Dp140 and Dp71 have been implicated in neuronal abnormalities in humans with DMD (Bardoni et al., 2000; Bardoni et al., 1999; Daoud et al., 2009a; Daoud et al., 2009b; Felisari et al., 2000; Moizard et al., 1998; Moizard et al., 2000; Pane et al., 2012; Taylor et al., 2010). Among all DMD models, only *mdx^3cv^* and *mdx βgeo* mice do not express Dp140 and Dp70. Surprisingly, neurocognitive behaviors of *mdx^3cv^* mice are only slightly different from those of *mdx* mice (Muntoni et al., 1991; Vaillend et al., 1998; Vaillend et al., 2004; Vaillend et al., 1995; Vaillend and Ungerer, 1999; Yamamoto et al., 2010). Dp71-specific knockout mice have also been generated and, interestingly, they show more severe learning impairment than *mdx* mice (Daoud et al., 2009b; Sarig et al., 1999). It is very likely that none of the existing mouse models can fully recapitulate the neurocognitive impairments of humans with DMD (D’Angelo and Bresolin, 2006). Nevertheless, most investigators have used *mdx* mice to dissect the molecular and cellular consequences of dystrophin deficiency in the brain. Collectively, these studies have revealed abnormalities in the hippocampus and in several other regions of the brain (Ghedini et al., 2012; Graciotti et al., 2008; Miranda et al., 2011; Miranda et al., 2009; Parames et al., 2014; Vaillend et al., 2004; Vaillend et al., 1999). So far, only exon skipping has been explored to treat CNS defects. Vaillend and colleagues injected an AAV exon-skipping vector to the *mdx* brain and found improvement of hippocampus function (Dallérac et al., 2011; Vaillend et al., 2010). Sekiguchi et al. ameliorated the abnormal freezing response (see Box 1) seen in *mdx* mice by injecting PMO AON to the ventricles of the brain (Sekiguchi et al., 2009). Utrophin has been considered as a highly promising replacement for dystrophin (see next section for details). Interestingly, a recent study suggested that utrophin upregulation in the brain might not rescue behavioral deficiency in *mdx* mice (Perronnet et al., 2012).

## Dystrophin-independent gene therapy for DMD: lessons from animal models

The striking phenotypic differences between dystrophin-deficient mice and affected humans have stimulated much interest in identifying the genes that modify DMD phenotypes. Compared with dystrophin-based therapy, the modulation of genes that already exist in the body has clear immunological advantages; the therapeutic expression of these genes is unlikely to induce immune rejection because they are considered as self (Ebihara et al., 2000).

Utrophin and α7β1-integrin are among the most obvious candidates to consider because: (1) similarly to dystrophin, they strengthen the sarcolemma by cross-linking the ECM and the cytoskeleton; (2) their expression is upregulated in *mdx* mice; (3) genetic elimination of either gene aggravates dystrophic manifestations in *mdx* mice; and (4) overexpression of either gene ameliorates muscle disease in *mdx* mice (Burkin et al., 2005; Burkin et al., 2001; Deconinck et al., 1997a; Deconinck et al., 1997b; Grady et al., 1997; Guo et al., 2006; Rafael et al., 1998; Rooney et al., 2006; Tinsley et al., 1998; Tinsley et al., 1996). As a result, gene therapy studies have been conducted in dystrophic mice (and some dogs) using full-length utrophin (Deol et al., 2007), mini-utrophin (Cerletti et al., 2003; Gilbert et al., 1999; Wakefield et al., 2000), micro-utrophin (Odom et al., 2008) and α7-integrin (Heller et al., 2013). As predicted from knockout and transgenic experiments, the dystrophic phenotype was significantly reduced by utrophin or integrin gene therapy.

Myostatin inhibition is another example of dystrophin-independent therapy for DMD. Myostatin is an endogenous muscle-growth inhibitor (Lee, 2004; McPherron et al., 1997). Mutations in the myostatin gene cause hypermuscularity in mouse, cattle, sheep, dog and humans (Stinckens et al., 2011). Elimination of the myostatin gene protects *mdx* mice by reducing fibrosis and increasing muscle strength (Wagner et al., 2002). These observations provide compelling justification to explore myostatin inhibition gene therapy in animal models and, more recently, in BMD patients (Mendell et al., 2015; Rodino-Klapac et al., 2009).

Evidence from preclinical studies is opening up new lines of investigation concerning how other endogenous genes could be used in DMD gene therapy. These include genes encoding cytotoxic T-cell GalNAc transferase (Xu et al., 2007), nNOS (Lai et al., 2014), sarcoplasmic reticulum calcium ATPase 2a (Shin et al., 2011a), peroxisome proliferator-activated receptor gamma coactivator 1-alpha (Selsby et al., 2012) and sarcospan (Marshall et al., 2013).

## Conclusions and perspective

Animal models have greatly enriched our understanding of the biological function of dystrophin and the pathology of DMD, providing excellent platforms for investigating the efficacy and toxicity of experimental gene therapies. Considerable progress has been made in model development in the last three decades. We now have a large (and still expanding) collection of animal models (supplementary material Table S1). Although this offers an unprecedented opportunity for cross-species comparison and translation (Poussin et al., 2014), it also adds complexity and difficulty in model selection for preclinical studies. The advantages and limitations of each model system can vary depending on the study question. Some aspects of the DMD pathology (such as neurocognitive deficiency) remain difficult to model. Furthermore, animals are not humans. The findings from animal studies may guide but not completely predict the outcome of clinical studies. Nevertheless, the value of animal models should never be underestimated. The development of an effective gene therapy for DMD has relied heavily, and will continue to rely, on animal models (Duan, 2011). Animal studies not only establish the proof-of-principle, they are also crucial for protocol optimization before and during human tests. Certain studies that cannot be performed in affected individuals (such as necropsy, *in situ* and *ex vivo* single-muscle force measurement) will have to be carried out in animal models. The field has surmounted many obstacles in the development of DMD models. The mild *mdx* mice are now complemented by numerous background and mutation variants that can better mimic affected humans. However, as therapies that have been in development for the last decade enter clinical trials, new questions are emerging. Many of these new questions (such as the immune response to the AAV vector and scaling-up of systemic gene transfer) might be better answered with cDMD dogs, a model that remains to be fully characterized (Duan, 2011; Duan, 2015).

## Supplementary Material

Supplementary Material
