# Supplementary material for: Animal models of Duchenne muscular dystrophy: from basic mechanisms to gene therapy
Source: Dis Model Mech. 2015 Mar;8(3):195–213. doi: 10.1242/dmm.018424 (PMC4348559; doi:10.1242/dmm.018424)
Supplement: Supplementary Material [file supp_8_3_195__index.html]

Supplementary Material 

# Animal models of Duchenne muscular dystrophy: from basic mechanisms to gene therapy

## DMM018424 Supplementary Material

**Files in this Data Supplement:**

- **Supplementary Material**
