# Supplementary material for: Animal models of Duchenne muscular dystrophy: from basic mechanisms to gene therapy
Source: Dis Model Mech. 2015 Mar;8(3):195–213. doi: 10.1242/dmm.018424 (PMC4348559; doi:10.1242/dmm.018424)
Supplement: Supplementary Material [file supp_8.3.195_DMM018424.pdf]

Supplementary Table 1. Animal models for DMD

Non-mammalian	Mutation	Comments	Reference
C. elegans		Various models available.	Reviewed in Chamberlain and Benian. 2000
Drosophila		Various models available.	Reviewed in Lloyd and Taylor, 2010
Zebrafish		Dystrophin-null sapje model has served as an excellent high-throughput system for drug screening.	Reviewed in Kunkel et al., 2006; Berger and Currie, 2012
**Murine***	**Mutation**	**Comments**	**Reference**
**Dystrophin-deficient mice**			
Mdx	Exon 23 point mutation	Most widely used model. On the C57BL/10 background. Available from the Jackson Laboratory (JL#001801).	Bulfield et al., 1984
Albino Mdx	Same as mdx	Mdx on the Albino background.	Krivov et al, 2009
Mdx/BALB/c	Same as mdx	Mdx on the BALB/c background.	Schmidt et al., 2011
Mdx/BL6	Same as mdx	Mdx on the C57BL/6 background. This strain has been used to generate IL-10/dystrophin dko mice (Nitahara-Kasahara et al., 2014) and myostatin/dystrophin dko mice (Wagner et al., 2002).	Duan et al., unpublished
Mdx/C3H	Same as mdx	Mdx on the C3H background.	Schmidt et al., 2011
Mdx/DBA2	Same as mdx	Mdx on the DBA2 background. More severe dystrophic phenotype. Available from the Jackson Laboratory (JL#013141).	Fukada et al., 2010
Mdx/FVB	Same as mdx	Mdx on the FVB background.	Wasala et al., 2015
Mdx2cv	Intron 42 point mutation	Chemically induced mutation. On the C57BL/6 background. Fewer revertant fibers. Available from the Jackson Laboratory (JL#002388).	Chapman et al., 1989
Mdx3cv	Intron 65 point mutation	Chemically induced mutation. On the C57BL/6 background. All dystrophin isoforms are eliminated but a near-full-length dystrophin is expressed at ~5% of the wild type level. Available from the Jackson Laboratory (JL#002377).	Chapman et al., 1989
Mdx4cv	Exon 53 point mutation	Chemically induced mutation. On the C57BL/6 background. Fewer revertant fibers. Available from the Jackson Laboratory (JL#002378).	Chapman et al., 1989
Mdx5cv	Exon 10 point mutation	Chemically induced mutation. On the C57BL/6 background. Skeletal muscle disease is more severe. Available from the Jackson Laboratory (JL#002388).	Chapman et al., 1989

CRKHR1	Unsequenced, dystrophin deficiency confirmed by immunofluorescence staining	ENU chemically induced mutation on the C3H background, screened for and found to have an elevated CK, centrally nucleated myofibers and dystrophin deficiency.	Aigner et al., 2009
Mdx52	Exon 52 deletion	Targeted inactivation. On the C57BL/6 background. Hot-spot mutation.	Araki et al., 1997
Mdx βgeo	Insertion of the β-geo gene trap cassette in intron 63	LacZ replaced the CR and CT domain. All dystrophin isoforms are mutated. The full-length dystrophin-LacZ fusion protein is not detectable but Dp71-LacZ fusion protein can be detected.	Wertz and Füchtbauer., 1998
DMD-null	Entire DMD gene deletion	Generated by Cre-loxP technology.	Kudoh et al., 2005
Dp71-null	Insertion of a β-geo cassette in intron 62. It disrupts Dp71 unique exon 1	Selective elimination of Dp71. Dp71 promoter driven LacZ expression. Similar LacZ expression pattern as mdx βgeo but muscle is not dystrophic. Dp71 deficiency is associated with early cataract formation in mice.	Sarig et al., 1999; Fort et al., 2014
Dup2	Exon 2 duplication	The only duplication mutation model. On the C57Bl/6 background.	Wein et al., 2014
**Immune deficient mdx mice**			
NSG-mdx4cv	Prkdc and IL2rg double deficient on the mdx4cv background	B cell, T cell and NK cell deficient. Innate immunity deficient. Multiple cytokine signaling pathway deficient. NSG mice are available from the Jackson Laboratory (JL#005557).	Arpke et al., 2013
$Rag2^{-}Il2rb^{-}Dmd^{-}$	Rag2 and IL2rb double deficient on the mdx βgeo background	B cell, T cell and NK cell deficient. Multiple cytokine signaling pathway deficient. No revertant fiber. Rag2/Il2rb double knock out strain is available from Taconic (#4111).	Bencze et al., 2012; Vallese et al., 2013
Scid mdx	DNA-dependent protein kinase catalytic subunit deficient (prkdc) on the mdx background	B cell and T cell deficient. Available from the Jackson Laboratory (JL#018018).	Farini et al., 2007
W41 mdx	C-kit receptor deficient on the mdx background	Haematopoietic deficient. Good for study bone marrow cell therapy in the absence of myeloablation by irradiation.	Walsh et al., 2011
**Phenotypic dko mice**			
α7/dystrophin dko or mdx/$\alpha7^{-/-}$	α7-Integrin/dystrophin double deficient	Severe dystrophic phenotype. Two independent lines exist. One is generated by Mayer and colleagues. The other is generated in the Burkin lab.	Rooney et al., 2006; Guo et al., 2006
$Adbn^{-/-}$ mdx	α-Dystrobrevin/dystrophin double deficient	Severe dystrophic phenotype.	Grady et al., 1999
Cmah-mdx	Cmah/dystrophin double deficient	Severe dystrophic phenotype. Humanized model. Available from the the Jackson Laboratory (JL#017929).	Chandrasekharan et al., 2010
d-Dko	δ-Sarcoglycan/dystrophin double deficient	Severe dystrophic phenotype.	Li et al., 2009

Desmin$^{-/-}$ mdx4cv	Desmin/dystrophin double deficient	Severe dystrophic phenotype.	Banks et al., 2014
Dmd^{mdx}/Large^{myd}	like-glycosyltransferase (LARGE)/dystrophin double deficient	Severe dystrophic phenotype.	Martins et al., 2013
DMD-null; Adam8$^{-/-}$	ADAM8 deficient and entie DMD gene deletion	This mouse is on the DMD-null background (Kudoh et al., 2005). ADAM8 deficiency hinders invasion of neutrophils into the damage myofiber. As a consequence, injured myofibers are not efficiently removed in dystrophin-null muscle.	Nishimura et al., 2014
Dysferlin/dystrophin dko	Dysferin/dystrophin double deficient	Severe dystrophic phenotype. Two independent lines exist. One is a cross between naturally occurring dysferlin-null A/J mice and mdx5cv mice. The other is a cross between dysferlin knockout mice and mdx mice.	Han et al., 2011; Hosur et al ., 2012
IL-10$^{-/-}$/mdx	Interleukin-10/dystrophin double deficient	Severe dystrophic phenotype. Prominent cardiomyopathy.	Nitahara-Kasahara et al., 2014
mdx/mTR	Telomerase RNA/dystrophin double deficient	Severe dystrophic phenotype. Two strains available at the Jackson Laboratory. One is on the mdx4cv background (JL#023535). The other is on the mdx background (JL#018915).	Sacco et al., 2010
mdx:MyoD $^{-/-}$	MyoD/dystrophin double deficient	Severe dystrophic phenotype. MyoD is only expressed in skeletal muscle. Interestingly, dko mice show severe dilated cardiomyopathy.	Megeney et al., 1996
mdx:utrophin $^{-/-}$ (Grady strain) or mdx/utrophin $^{-/-}$ (Deconinck strain)	Utrophin/dystrophin double deficient	Severe dystrophic phenotype. Two independent strains exist. Both are available at the Jackson Laboratory. In the Grady strain (Utrntm1Jrs Dmdmdx), all utrophin isoforms are inactivated by a targeted mutation at the utrophin CR domain (JL#016622). In the Deconnick strain (Utrntm1Ked Dmdmdx), only the largest utrophin isoform is inactivated by a targeted mutation at utrophin exon 7 (JL#014563).	Deconinck et al., 1997; Grady et al., 1997
PAI-1$^{-/-}$ mdx	Plasminogen activator inhibitor-1 (PAI-1)/dystrophin double deficient	Dko mice show early onset fibrosis and higher CK than mdx.	Ardite et al., 2012
**Dko mice with phenotype similar to mdx**			
msDKO	Cytosolic γ-actin/dystrophin double deficient	Phenotype similar to that of mdx.	Prins et al., 2008
iNOS-null mdx or iNOS/Dys DKO	iNOS/dystrophin double deficient	Phenotype similar to that of mdx. Two independent strains exist. The Tidball lab stain is on the mdx background. The Duan lab strain is on the mdx4cv background.	Villalta et al., 2009; Li et al., 2011a
PVKO-mdx	Parvalbumin/dystrophin double deficient	Phenotype similar to that of mdx.	Raymackers et al., 2003
**Dko mice with reduced disease**			

cIAP1$^{-/-}$;mdx	Cellular inhibitor of apoptosis 1 (cIAP1)/dystrophin double deficient	Reduced disease. Soleus pathology reduced. Diaphragm function improved.	Enwere et al., 2013
Fib$^{-/-}$mdx	Fibrinogen/dystrophin double deficient	Reduced disease. Inflammation and degeneration reduced. Regeneration, grip strength and treadmill improved.	Vidal et al., 2012
Finp1$^{-/-}$mdx4CV	Folliculin interacting protein-1 (Fnip1) deficint mice on the mdx4cv background.	Disease reduced due to Finp1-deficiency associated switch to type I fiber. Central nucleation and the CK level are reduced. Membrane integrity improved.	Reyes et al., 2014 December 29 (online publication ahead of print)
Mdx-casp	Caspase-12/dystrophin double deficient	Reduced disease. Muscle force improved. Myofiber degeneration reduced but central nucleation, CK and fibrosis not changed.	Moorwood and Barton et al., 2014
mdx/Mkp5$^{-/-}$	Mitogen-activated protein kinases phosphatase-5 (Mkp5)/dystrophin double deficient	Reduced disease. Reduced degeneration, CK. Improved regeneration, grip strength and EDL force.	Shi et al., 2013
mdx/myd88$^{-/-}$	Myeloid differentiation primary response protein 88 (myd88)/dystrophin double deficient	Reduced disease. Skeletal muscle disease is reduced in 12-m-old mice but not in 2 to 4-m-old mice. Heart disease is reduced in 10 to 12-m-old mice.	Henriques-Pons et al., 2014
mdx/q$^{-/-}$	Protein kinase C q (PKCq)/dystrophin double deficient	Reduced disease. Reduced degeneration and inflammation. Improved regeneration and treadmill performance.	Madaro et al, 2012
mdx/sgk1$^{-/-}$	Serum-and glucocorticoid-induced kinase 1 (sgk1) and dystrophin double deficient	Reduced disease. Improved specific force, muscle fatigueability, and histology. Normalization of fibrosis.	Steinberger et al., 2014
mdx-Xist$^{\Delta hs}$	Xist/dystrophin double knockout	Variable level of dystrophin expression as low as 5%.	van Putten et al., 2013
Mstn$^{-/-}$/mdx	Myostatin/dystrophin double deficient	Reduced disease. Limb muscle is more muscular and stronger. Diaphragm fibrosis is reduced.	Wagner et al 2002
OPN DMM	Osteopontin (OPN)/dystrophin double deficient	Reduced disease. Improved regeneration and grip strength. Reduced inflammation and fibrosis.	Vetrone et al., 2009
**Transgenic mdx mice**			
Full-length dystrophin transgenic mdx	Transgenic over-expression of full-length dystrophin in the mdx background	Multiple lines were generated by different labs. All show protection. 50-fold over-expression is not toxic to skeletal muscle.	Cox et al., 1993; Phelps et al., 1995; Wells et al., 1995
Dp71 transgenic mdx	Transgenic over-expression of Dp71 in the mdx background	More severe disease confirmed by two independent lines made in two different labs.	Cox et al., 1994; Greenberg et al., 1994
Dp116 transgenic mdx4cv	Transgenic over-expression of Dp116 in the mdx4cv background	More severe disease.	Judge et al., 2006
Dp116:mdx:utrophin $^{-/-}$	Transgenic over-expression of Dp116 in the utrophin/dystrophin dko background	Improved growth, mobility and lifespan but no change in histopathology, specific force and CK.	Judge et al., 2011

Dp260 transgenic mdx	Transgenic over-expression of Dp260 in the mdx background	Reduced but not completely prevented histopathology. Improved resistance to eccentric contraction injury but did not improve specific force.	Warner et al., 2002
Dp260 in mdx/utrn$^{-/-}$	Transgenic over-expression of Dp260 in the utrophin/dystrophin dko background	Severe lethal phenotype is converted to mild myopathy.	Gaedigk et al., 2006
Δ17-48 transgenic mdx	Transgenic over-expression of the naturally occurring Δ17-48 mini-dystrophin gene in the mdx background	Two independent lines were generated. Both showed muscle protection.	Phelps et al., 1995; Wells et al., 1992 and 1995
ΔH2-R19 transgenic mdx	Transgenic over-expression of the synthetic ΔH2-R19 mini-dystrophin gene in the mdx background	Completely reduced histopathology and normalized muscle force but did not restore sarcolemmal nNOS.	Harper et al., 2002
Cardiac-specific ΔH2-R19 transgenic mdx	Transgenic over-expression of the synthetic ΔH2-R19 mini-dystrophin gene in the heart of mdx mice	Effectively protected but did not fully normalize the heart.	Bostick et al., 2009
ΔH2-R15 transgenic mdx	Transgenic over-expression of the synthetic ΔH2-R15 mini-dystrophin gene in the mdx background	Complete correction of the dystrophic phenotype including nNOS and functional ischemia.	Lai et al., 2009; Hakim and Duan 2013
Micro-dystrophin transgenic	Transgenic over-expression of various synthetic micro-dystrophin genes in the mdx background	Many lines are established for different microgenes. ΔR4-23 and ΔR4-23/C yield excellent protection but they don't restore nNOS. Hinge 2 in these two microgenes compromises function. ΔR2-15/R18-19/R20-23/C contains hinge 3 and is the only microgene capable of restoring nNOS.	Harper et al., 2002; Li et al., 2011b; Sakamoto et al., 2002, Hakim et al., 2013; Wang et al., 2008; Ferrer et al 2004
Fiona	Transgenic over-expression of full-length utrophin in the mdx background	Excellent protection but does not restore nNOS.	Tinsley et al., 1998; Li et al., 2010
Laminin α1 transgenic mdx	Transgenic over-expression of the laminin α1 chain in the mdx background	Used to study laminin-111 protein therapy. Phenotype appeared to be very similar to mdx, without any benefit or harm.	Gawlik et al., 2011
**Canine**	**Mutation**	**Comments**	**References**
Alaskan malamute dystrophic dog	Unknown but dystrophin deficiency is confirmed	Case report.	Ito et al., 2011
CKCS-MD	Intron 50 point mutation resulting in exon 50 exclusion from the mRNA	Spontaneous mutation in the Cavelier King Charles Spaniel (CKCS) breed. Small breed. Hot-spot mutation. Colony maintained at Royal Veterinary College, UK.	Walmsley et al., 2010
Cocker spaniel dystrophic dog	Deletion of four nucleotides in exon 65	No colony established.	Kornegay et al., 2012

CXMDj	Same as GRMD	GRMD crossed to the beagle background. Small breed. Reduced phenotype. Colony maintained at the National Center of Neurology and Psychiatry, Japan.	Shimatsu et al., 2003
GLRMD	Same as GRMD	Hybrid background of golden retriever and Labrador retriever.	Miyazato et al., 2011
Grand Basset Griffon Vendeen dystrophic dog	Unknown but dystrophin deficiency is confirmed	Case report.	Klarenbeek et al., 2007
GRMD	Intron 6 point mutation resulting in the exclusion of exon 7 from the mRNA	Spontaneous mutation in the golden retriever (GR) breed. Similar disease as human patients. Most widely used dog model. Multiple colonies exist worldwide.	Valentine et al., 1986; Cooper et al., 1988; Kornegay et al., 1988
GSHP MD	Whole gene deletion	Spontaneous mutation in the German short haired pointer (GSHP) breed.	Schatzberg et al., 1999
Hybrid cDMD dogs with mixed genetic background and multiple mutations	Various	Generated by artificial insemination by crossing different cDMD breeds. Resembles genetic diversity seen in human patients.	Fine et al., 2011; Miyazato et al., 2011; Shin et al., 2013a; Shin et al 2013b; Yang et al 2012;
Japanese spitz dystrophic dog	Inversion between intron 19 of dystrophin gene and retinitis pigmentosa GTPase regulator gene (RPGN)	Case report.	Jones et al., 2004; Atencia-Fernandez et al., 2015
Labrador Retriever BMD dog	Unknown	Case report. Low-level uniform expression of a ~135 kDa dystrophin protein. Mild phenotype. This is the only reported BMD dog case.	Baroncelli et al., 2014
Labrador Retriever dystrophic dog	Unknown but dystrophin deficiency is confirmed	Case report.	Bergman et al., 2002
Labrador Retriever dystrophic dog	Repetitive element insertion in intron 19	Spontaneous mutation. Colony maintained at the University of Missouri and Auburn University.	Smith et al., 2007
Lurcher dystrophic dog	Unknown but dystrophin deficiency is confirmed	Case report of two pups in the same litter. Possible response to L-carnitine supplementation in one of the pups.	Giannasi et al., 2015
Miniature schnauzer dystrophic dog	Unknown but dystrophin deficiency is confirmed	Case report.	Paola et al., 1993
Norfolk Terrier dystrophy	Unknown but dystrophin deficiency is confirmed	No colony established.	Beltran et al., 2014
Old English sheepdog dystrophic dog	Unknown but dystrophin deficiency is confirmed	Case report.	Wieczorek et al., 2006
Rat terrier dystrophic dog	Unknown but dystrophin deficiency is confirmed	Case report. Unusual hypertrophic presentation in the cervical and proximal limb muscles.	Wetterman et al., 2000
Rotteweiler dystrophic dog	Nonsense point mutation in exon 58	No colony established.	Kornegay et al., 2012; Winand et al 1994b

Tibetan terrier dystrophic dog	Exons 8-29 deletion	No colony established.	Kornegay et al., 2012
Weimaraner dystrophic dog	Unknown but dystrophin deficiency is confirmed	Case report.	Baltzer et al., 2007
Welsh Corgi MD	LINE-1 insertion in intron 13	Spontaneous mutation. Colony maintained at the University of Missouri and Auburn University.	Smith et al., 2011

**Other Mammalian**	**Mutation**	**Comments**	**References**
DMD rat #1	Exon 3-6 deletion using the CRISPR/Cas technology	New model.	Nakamura et al., 2014
DMD rat #2	Frame shifting 11 bp deletion in exon 23 using TALEN technology, creates premature stop codon	New model. 5% revertant fiber expression. More severe skeletal muscle fibrosis than *mdx* . Fibrotic lesions in myocardium, though showed concentric hypertrophy rather than eccentric.	Larcher et al., 2014
DMD cat #1	Dp427 promoter and exon 1 deletion	Spontaneous mutation. Prominent muscle hypertrophy. Independent cases have been reported in USA and UK.	Winand et al., 1994a; Carpenter et al., 1989; Blunden and Gower., 2011
DMD cat #2	Similar but not identical deletion as in DMD cat #1	Spontaneous mutation. Primary symptom is regurgitation due to megaesophagus. However, there is no muscle hypertrophy.	Gambino et al., 2014
BMD pig	Exon 41 missense mutation (changing arginine to tryptophan)	Spontaneous mutation. Dystrophin expression is reduced to ~30% of normal. The primary clinical manefestation is stress-induced sudden death. Minimum dystrophic symptom.	Nonneman et al., 2012
DMD Pig #1	Engineered deletion of exon 52	Hot-spot deletion. Marked utrophin upregulation.	Klymiuk et al., 2013
DMD Pig #2	Cre-LoxP engineered deletion of exon 52.	Hot-spot deletion.	Rogers and Swart., 2014

*, The name of the mouse model is according to the first publication that described the model.

Reference list for Supplementary Table 1

Aigner, B., Rathkolb, B., Klaften, M., Sedlmeier, R., Klempt, M., Wagner, S., Michel, D., Mayer, U., Klopstock, T., de Angelis, M. H. et al. (2009). Generation of N-ethyl-N-nitrosourea-induced mouse mutants with deviations in plasma enzyme activities as novel organ-specific disease models. *Exp Physiol* **94**, 412-21.

Araki, E., Nakamura, K., Nakao, K., Kameya, S., Kobayashi, O., Nonaka, I., Kobayashi, T. and Katsuki, M. (1997). Targeted disruption of exon 52 in the mouse dystrophin gene induced muscle degeneration similar to that observed in Duchenne muscular dystrophy. *Biochem Biophys Res Commun* **238**, 492-7.

Ardite, E., Perdiguero, E., Vidal, B., Gutarra, S., Serrano, A. L. and Munoz-Canoves, P. (2012). PAI-1-regulated miR-21 defines a novel age-associated fibrogenic pathway in muscular dystrophy. *J Cell Biol* **196**, 163-75.

Arpke, R. W., Darabi, R., Mader, T. L., Zhang, Y., Toyama, A., Lonetree, C. L., Nash, N., Lowe, D. A., Perlingeiro, R. C. and Kyba, M. (2013). A new immuno-, dystrophin-deficient model, the NSG-mdx(4Cv) mouse, provides evidence for functional improvement following allogeneic satellite cell transplantation. *Stem Cells* **31**, 1611-20.

Atencia-Fernandez, S., Shiel, R.E., Mooney, C.T., Nolan, C.M. (2015). Muscular dystrophy in the Japanese Spitz: an inversion disrupts the *DMD* and *RPGR* genes. *Anim Genet.* [Epub ahead of print] doi: 10.1111/age. 12266.

Baltzer, W. I., Calise, D. V., Levine, J. M., Shelton, G. D., Edwards, J. F. and Steiner, J. M. (2007). Dystrophin-deficient muscular dystrophy in a Weimaraner. *J Am Anim Hosp Assoc* **43**, 227-32.

Banks, G. B., Combs, A. C., Odom, G. L., Bloch, R. J. and Chamberlain, J. S. (2014). Muscle structure influences utrophin expression in mdx mice. *PLoS Genet* **10**, e1004431.

Baroncelli, A. B., Abellonio, F., Pagano, T. B., Esposito, I., Peirone, B., Papparella, S. and Paciello, O. (2014). Muscular dystrophy in a dog resembling human becker muscular dystrophy. *J Comp Pathol* **150**, 429-33.

Beltran, E., Shelton, G. D., Guo, L. T., Dennis, R., Sanchez-Masian, D., Robinson, D. and De Risio, L. (2014). Dystrophin-deficient muscular dystrophy in a Norfolk terrier. *J Small Anim Pract*, 2014 Oct 29. doi: 10.1111/jsap.12292. [Epub ahead of print].

Bencze, M., Negroni, E., Vallese, D., Yacoub-Youssef, H., Chaouch, S., Wolff, A., Aamiri, A., Di Santo, J. P., Chazaud, B., Butler-Browne, G. et al. (2012). Proinflammatory macrophages enhance the regenerative capacity of human myoblasts by modifying their kinetics of proliferation and differentiation. *Mol Ther* **20**, 2168-79.

Berger, J. and Currie, P. D. (2012). Zebrafish models flex their muscles to shed light on muscular dystrophies. *Dis Model Mech* **5**, 726-32.

Bergman, R. L., Inzana, K. D., Monroe, W. E., Shell, L. G., Liu, L. A., Engvall, E. and Shelton, G. D. (2002). Dystrophin-deficient muscular dystrophy in a Labrador retriever. *J Am Anim Hosp Assoc* **38**, 255-61.

Blunden, A. S. and Gower, S. (2011). Hypertrophic feline muscular dystrophy: diagnostic overview and a novel immunohistochemical diagnostic method using formalin-fixed tissue. *Vet Rec* **168**, 510.

Bostick, B., Yue, Y., Long, C., Marschalk, N., Fine, D. M., Chen, J. and Duan, D. (2009). Cardiac expression of a mini-dystrophin that normalizes skeletal muscle force only partially restores heart function in aged Mdx mice. *Mol Ther* **17**, 253-61.

Bulfield, G., Siller, W. G., Wight, P. A. and Moore, K. J. (1984). X chromosome-linked muscular dystrophy (mdx) in the mouse. *Proc Natl Acad Sci U S A* **81**, 1189-92.

Carpenter, J. L., Hoffman, E. P., Romanul, F. C., Kunkel, L. M., Rosales, R. K., Ma, N. S., Dasbach, J. J., Rae, J. F., Moore, F. M., McAfee, M. B. et al. (1989). Feline muscular dystrophy with dystrophin deficiency. *Am J Pathol* **135**, 909-19.

Chamberlain, J. S. and Benian, G. M. (2000). Muscular dystrophy: the worm turns to genetic disease. *Curr Biol* **10**, R795-7.

Chandrasekharan, K., Yoon, J. H., Xu, Y., deVries, S., Camboni, M., Janssen, P. M., Varki, A. and Martin, P. T. (2010). A human-specific deletion in mouse Cmah increases disease severity in the mdx model of Duchenne muscular dystrophy. *Sci Transl Med* **2**, 42ra54.

Chapman, V. M., Miller, D. R., Armstrong, D. and Caskey, C. T. (1989). Recovery of induced mutations for X chromosome-linked muscular dystrophy in mice. *Proc Natl Acad Sci U S A* **86**, 1292-6.

Cooper, B. J., Winand, N. J., Stedman, H., Valentine, B. A., Hoffman, E. P., Kunkel, L. M., Scott, M. O., Fischbeck, K. H., Kornegay, J. N., Avery, R. J. et al. (1988). The homologue of the Duchenne locus is defective in X-linked muscular dystrophy of dogs. *Nature* **334**, 154-6.

Cox, G. A., Cole, N. M., Matsumura, K., Phelps, S. F., Hauschka, S. D., Campbell, K. P., Faulkner, J. A. and Chamberlain, J. S. (1993). Overexpression of dystrophin in transgenic mdx mice eliminates dystrophic symptoms without toxicity [see comments]. *Nature* **364**, 725-9.

Cox, G. A., Sunada, Y., Campbell, K. P. and Chamberlain, J. S. (1994). Dp71 can restore the dystrophin-associated glycoprotein complex in muscle but fails to prevent dystrophy. *Nat Genet* **8**, 333-9.

Deconinck, A. E., Rafael, J. A., Skinner, J. A., Brown, S. C., Potter, A. C., Metzinger, L., Watt, D. J., Dickson, J. G., Tinsley, J. M. and Davies, K. E. (1997). Utrophin-dystrophin-deficient mice as a model for Duchenne muscular dystrophy. *Cell* **90**, 717-27.

Enwere, E. K., Boudreault, L., Holbrook, J., Timusk, K., Earl, N., LaCasse, E., Renaud, J. M. and Korneluk, R. G. (2013). Loss of cIAP1 attenuates soleus muscle pathology and improves diaphragm function in mdx mice. *Hum Mol Genet* **22**, 867-78.

Farini, A., Meregalli, M., Belicchi, M., Battistelli, M., Parolini, D., D'Antona, G., Gavina, M., Ottoboni, L., Constantin, G., Bottinelli, R. et al. (2007). T and B lymphocyte depletion has a marked effect on the fibrosis of dystrophic skeletal muscles in the scid/mdx mouse. *J Pathol* **213**, 229-38.

Ferrer, A., Foster, H., Wells, K. E., Dickson, G. and Wells, D. J. (2004). Long-term expression of full-length human dystrophin in transgenic mdx mice expressing internally deleted human dystrophins. *Gene Ther* **11**, 884-93.

Fine, D. M., Shin, J. H., Yue, Y., Volkmann, D., Leach, S. B., Smith, B. F., McIntosh, M. and Duan, D. (2011). Age-matched comparison reveals early

electrocardiography and echocardiography changes in dystrophin-deficient dogs. *Neuromuscul Disord* **21**, 453-61.

Fort, P. E., Darche, M., Sahel, J. A., Rendon, A. and Tadayoni, R. (2014). Lack of dystrophin protein Dp71 results in progressive cataract formation due to loss of fiber cell organization. *Mol Vis* **20**, 1480-90.

Fukada, S., Morikawa, D., Yamamoto, Y., Yoshida, T., Sumie, N., Yamaguchi, M., Ito, T., Miyagoe-Suzuki, Y., Takeda, S., Tsujikawa, K. et al. (2010). Genetic background affects properties of satellite cells and mdx phenotypes. *Am J Pathol* **176**, 2414-24.

Gaedigk, R., Law, D. J., Fitzgerald-Gustafson, K. M., McNulty, S. G., Nsumu, N. N., Modrcin, A. C., Rinaldi, R. J., Pinson, D., Fowler, S. C., Bilgen, M. et al. (2006). Improvement in survival and muscle function in an mdx/utrn(-/-) double mutant mouse using a human retinal dystrophin transgene. *Neuromuscul Disord* **16**, 192-203.

Gambino, A. N., Mouser, P. J., Shelton, G. D. and Winand, N. J. (2014). Emergent presentation of a cat with dystrophin-deficient muscular dystrophy. *J Am Anim Hosp Assoc* **50**, 130-5.

Gawlik, K. I., Oliveira, B. M. and Durbeej, M. (2011). Transgenic expression of Laminin alpha1 chain does not prevent muscle disease in the mdx mouse model for Duchenne muscular dystrophy. *Am J Pathol* **178**, 1728-37.

Giannasi, C., Tappin, S.W., Guo, L.T., Shelton, G.D., Palus, V. (2015). Dystrophin-deficient muscular dystrophy in two lurcher siblings. *J Small Anim Pract* [Epub ahead of print] doi: 10.1111/jsap.12331.

Grady, R. M., Grange, R. W., Lau, K. S., Maimone, M. M., Nichol, M. C., Stull, J. T. and Sanes, J. R. (1999). Role for alpha-dystrobrevin in the pathogenesis of dystrophin-dependent muscular dystrophies. *Nat Cell Biol* **1**, 215-20.

Grady, R. M., Teng, H., Nichol, M. C., Cunningham, J. C., Wilkinson, R. S. and Sanes, J. R. (1997). Skeletal and cardiac myopathies in mice lacking utrophin and dystrophin: a model for Duchenne muscular dystrophy. *Cell* **90**, 729-38.

Greenberg, D. S., Sunada, Y., Campbell, K. P., Yaffe, D. and Nudel, U. (1994). Exogenous Dp71 restores the levels of dystrophin associated proteins but does not alleviate muscle damage in mdx mice. *Nat Genet* **8**, 340-4.

Guo, C., Willem, M., Werner, A., Raivich, G., Emerson, M., Neyses, L. and Mayer, U. (2006). Absence of alpha7 integrin in dystrophin-deficient mice causes a myopathy similar to Duchenne muscular dystrophy. *Hum Mol Genet* **15**, 989-98.

Hakim, C. H. and Duan, D. (2013). Truncated dystrophins reduce muscle stiffness in the extensor digitorum longus muscle of mdx mice. *J Appl Physiol* **114**, 482-9.

Han, R., Rader, E. P., Levy, J. R., Bansal, D. and Campbell, K. P. (2011). Dystrophin deficiency exacerbates skeletal muscle pathology in dysferlin-null mice. *Skelet Muscle* **1**, 35.

Harper, S. Q., Hauser, M. A., DelloRusso, C., Duan, D., Crawford, R. W., Phelps, S. F., Harper, H. A., Robinson, A. S., Engelhardt, J. F., Brooks, S. V. et al. (2002). Modular flexibility of dystrophin: implications for gene therapy of Duchenne muscular dystrophy. *Nat Med* **8**, 253-61.

Henriques-Pons, A., Yu, Q., Rayavarapu, S., Cohen, T. V., Ampong, B., Cha, H. J., Jahnke, V., Van der Meulen, J., Wang, D., Jiang, W. et al. (2014). Role of Toll-like receptors in the pathogenesis of dystrophin-deficient skeletal and heart muscle. *Hum*

Mol Genet **23**, 2604-17.

Hosur, V., Kavirayani, A., Riefler, J., Carney, L. M., Lyons, B., Gott, B., Cox, G. A. and Shultz, L. D. (2012). Dystrophin and dysferlin double mutant mice: a novel model for rhabdomyosarcoma. *Cancer Genet* **205**, 232-41.

Ito, D., Kitagawa, M., Jeffery, N., Okada, M., Yoshida, M., Kobayashi, M., Nakamura, A. and Watari, T. (2011). Dystrophin-deficient muscular dystrophy in an Alaskan malamute. *Vet Rec* **169**, 127.

Jones, B. R., Brennan, S., Mooney, C. T., Callanan, J. J., McAllister, H., Guo, L. T., Martin, P. T., Engvall, E. and Shelton, G. D. (2004). Muscular dystrophy with truncated dystrophin in a family of Japanese Spitz dogs. *J Neurol Sci* **217**, 143-9.

Judge, L. M., Arnett, A. L., Banks, G. B. and Chamberlain, J. S. (2011). Expression of the dystrophin isoform Dp116 preserves functional muscle mass and extends lifespan without preventing dystrophy in severely dystrophic mice. *Hum Mol Genet* **20**, 4978-90.

Judge, L. M., Haraguchi, M. and Chamberlain, J. S. (2006). Dissecting the signaling and mechanical functions of the dystrophin-glycoprotein complex. *J Cell Sci* **119**, 1537-46.

Klarenbeek, S., Gerritzen-Bruning, M. J., Rozemuller, A. J. and van der Lugt, J. J. (2007). Canine X-linked muscular dystrophy in a family of Grand Basset Griffon Vendeen dogs. *J Comp Pathol* **137**, 249-52.

Klymiuk, N., Blutke, A., Graf, A., Krause, S., Burkhardt, K., Wuensch, A., Krebs, S., Kessler, B., Zakhartchenko, V., Kurome, M. et al. (2013). Dystrophin-deficient pigs provide new insights into the hierarchy of physiological derangements of dystrophic muscle. *Hum Mol Genet* **22**, 4368-82.

Kornegay, J. N., Bogan, J. R., Bogan, D. J., Childers, M. K., Li, J., Nghiem, P., Detwiler, D. A., Larsen, C. A., Grange, R. W., Bhavaraju-Sanka, R. K. et al. (2012). Canine models of Duchenne muscular dystrophy and their use in therapeutic strategies. *Mamm Genome* **23**, 85-108.

Kornegay, J. N., Tuler, S. M., Miller, D. M. and Levesque, D. C. (1988). Muscular dystrophy in a litter of golden retriever dogs. *Muscle Nerve* **11**, 1056-64.

Krivov, L. I., Stenina, M. A., Yarygin, V. N., Polyakov, A. V., Savchuk, V. I., Obrubov, S. A. and Komarova, N. V. (2009). A new genetic variant of mdx mice: study of the phenotype. *Bull Exp Biol Med* **147**, 625-9.

Kudoh, H., Ikeda, H., Kakitani, M., Ueda, A., Hayasaka, M., Tomizuka, K. and Hanaoka, K. (2005). A new model mouse for Duchenne muscular dystrophy produced by 2.4 Mb deletion of dystrophin gene using Cre-loxP recombination system. *Biochem Biophys Res Commun* **328**, 507-16.

Kunkel, L. M., Bachrach, E., Bennett, R. R., Guyon, J. and Steffen, L. (2006). Diagnosis and cell-based therapy for Duchenne muscular dystrophy in humans, mice, and zebrafish. *J Hum Genet* **51**, 397-406.

Lai, Y., Thomas, G. D., Yue, Y., Yang, H. T., Li, D., Long, C., Judge, L., Bostick, B., Chamberlain, J. S., Terjung, R. L. et al. (2009). Dystrophins carrying spectrin-like repeats 16 and 17 anchor nNOS to the sarcolemma and enhance exercise performance in a mouse model of muscular dystrophy. *J. Clin. Invest.* **119**, 624-635.

Larcher, T., Lafoux, A., Tesson, L., Remy, S., Thepenier, V., Francois, V., Le Guiner, C., Goubin, H., Dutilleul, M., Guigand, L. et al. (2014). Characterization of

dystrophin deficient rats: a new model for Duchenne muscular dystrophy. *PLoS One* **9**, e110371.

Li, D., Bareja, A., Judge, L., Yue, Y., Lai, Y., Fairclough, R., Davies, K. E., Chamberlain, J. S. and Duan, D. (2010). Sarcolemmal nNOS anchoring reveals a qualitative difference between dystrophin and utrophin. *J Cell Sci* **123**, 2008-13.

Li, D., Long, C., Yue, Y. and Duan, D. (2009). Sub-physiological sarcoglycan expression contributes to compensatory muscle protection in mdx mice. *Hum Mol Genet* **18**, 1209-20.

Li, D., Shin, J. H. and Duan, D. (2011a). iNOS ablation does not improve specific force of the extensor digitorum longus muscle in dystrophin-deficient mdx4cv mice. *PLoS One* **6**, e21618.

Li, D., Yue, Y., Lai, Y., Hakim, C. H. and Duan, D. (2011b). Nitrosative stress elicited by nNOSmu delocalization inhibits muscle force in dystrophin-null mice. *J Pathol* **223**, 88-98.

Lloyd, T. E. and Taylor, J. P. (2010). Flightless flies: Drosophila models of neuromuscular disease. *Ann N Y Acad Sci* **1184**, e1-20.

Madaro, L., Pelle, A., Nicoletti, C., Crupi, A., Marrocco, V., Bossi, G., Soddu, S. and Bouche, M. (2012). PKC theta ablation improves healing in a mouse model of muscular dystrophy. *PLoS One* **7**, e31515.

Martins, P. C., Ayub-Guerrieri, D., Martins-Bach, A. B., Onofre-Oliveira, P., Malheiros, J. M., Tannus, A., de Sousa, P. L., Carlier, P. G. and Vainzof, M. (2013). Dmdmdx/Largemyd: a new mouse model of neuromuscular diseases useful for studying physiopathological mechanisms and testing therapies. *Dis Model Mech* **6**, 1167-74.

Megeney, L. A., Kablar, B., Garrett, K., Anderson, J. E. and Rudnicki, M. A. (1996). MyoD is required for myogenic stem cell function in adult skeletal muscle. *Genes Dev* **10**, 1173-83.

Miyazato, L. G., Moraes, J. R., Beretta, D. C. and Kornegay, J. N. (2011). Muscular dystrophy in dogs: does the crossing of breeds influence disease phenotype? *Vet Pathol* **48**, 655-62.

Moorwood, C. and Barton, E. R. (2014). Caspase-12 ablation preserves muscle function in the mdx mouse. *Hum Mol Genet* **23**,5325-41.

Nakamura, K., Fujii, W., Tsuboi, M., Tanihata, J., Teramoto, N., Takeuchi, S., Naito, K., Yamanouchi, K. and Nishihara, M. (2014). Generation of muscular dystrophy model rats with a CRISPR/Cas system. *Sci Rep* **4**, 5635.

Nishimura, D., Sakai, H., Sato, T., Sato, F., Nishimura, S., Toyama-Sorimachi, N., Bartsch, J. W. and Sehara-Fujisawa, A. (2014). Roles of ADAM8 in elimination of injured muscle fibers prior to skeletal muscle regeneration. *Mech Dev*.

Nitahara-Kasahara, Y., Hayashita-Kinoh, H., Chiyo, T., Nishiyama, A., Okada, H., Takeda, S. and Okada, T. (2014). Dystrophic mdx mice develop severe cardiac and respiratory dysfunction following genetic ablation of the anti-inflammatory cytokine IL-10. *Hum Mol Genet* **23**, 3990-4000.

Nonneman, D. J., Brown-Brandl, T., Jones, S. A., Wiedmann, R. T. and Rohrer, G. A. (2012). A defect in dystrophin causes a novel porcine stress syndrome. *BMC Genomics* **13**, 233.

Paola, J. P., Podell, M. and Shelton, G. D. (1993). Muscular dystrophy in a miniature Schnauzer. *Prog Vet Neurol* **4**, 14-8.

Phelps, S. F., Hauser, M. A., Cole, N. M., Rafael, J. A., Hinkle, R. T., Faulkner, J. A. and Chamberlain, J. S. (1995). Expression of full-length and truncated dystrophin mini-genes in transgenic mdx mice. *Hum Mol Genet* **4**, 1251-8.

Prins, K. W., Lowe, D. A. and Ervasti, J. M. (2008). Skeletal muscle-specific ablation of gamma(cyto)-actin does not exacerbate the mdx phenotype. *PLoS One* **3**, e2419.

Raymackers, J. M., Debaix, H., Colson-Van Schoor, M., De Backer, F., Tajeddine, N., Schwaller, B., Gailly, P. and Gillis, J. M. (2003). Consequence of parvalbumin deficiency in the mdx mouse: histological, biochemical and mechanical phenotype of a new double mutant. *Neuromuscul Disord* **13**, 376-87.

Reyes, N. L., Banks, G. B., Tsang, M., Margineantu, D., Gu, H., Djukovic, D., Chan, J., Torres, M., Liggitt, H. D., Hirenallur, S. D. et al. (2014). Fnip1 regulates skeletal muscle fiber type specification, fatigue resistance, and susceptibility to muscular dystrophy. *Proc Natl Acad Sci U S A*.

Rogers, C. S. and Swart, J. R. (2014). Animal Models of Duchenne Muscular Dystrophy, pp. 7. United States: Exemplar Genetics, LLC.

Rooney, J. E., Welser, J. V., Dechert, M. A., Flintoff-Dye, N. L., Kaufman, S. J. and Burkin, D. J. (2006). Severe muscular dystrophy in mice that lack dystrophin and alpha7 integrin. *J Cell Sci* **119**, 2185-95.

Sacco, A., Mourkioti, F., Tran, R., Choi, J., Llewellyn, M., Kraft, P., Shkreli, M., Delp, S., Pomerantz, J. H., Artandi, S. E. et al. (2010). Short Telomeres and Stem Cell Exhaustion Model Duchenne Muscular Dystrophy in mdx/mTR Mice. *Cell* **143**, 1059-71.

Sakamoto, M., Yuasa, K., Yoshimura, M., Yokota, T., Ikemoto, T., Suzuki, M., Dickson, G., Miyagoe-Suzuki, Y. and Takeda, S. (2002). Micro-dystrophin cDNA ameliorates dystrophic phenotypes when introduced into mdx mice as a transgene. *Biochem Biophys Res Commun* **293**, 1265-72.

Sarig, R., Mezger-Lallemand, V., Gitelman, I., Davis, C., Fuchs, O., Yaffe, D. and Nudel, U. (1999). Targeted inactivation of Dp71, the major non-muscle product of the DMD gene: differential activity of the Dp71 promoter during development. *Hum Mol Genet* **8**, 1-10.

Schatzberg, S. J., Olby, N. J., Breen, M., Anderson, L. V., Langford, C. F., Dickens, H. F., Wilton, S. D., Zeiss, C. J., Binns, M. M., Kornegay, J. N. et al. (1999). Molecular analysis of a spontaneous dystrophin 'knockout' dog. *Neuromuscul Disord* **9**, 289-95.

Schmidt, W. M., Uddin, M. H., Dysek, S., Moser-Thier, K., Pirker, C., Hoger, H., Ambros, I. M., Ambros, P. F., Berger, W. and Bittner, R. E. (2011). DNA damage, somatic aneuploidy, and malignant sarcoma susceptibility in muscular dystrophies. *PLoS Genet* **7**, e1002042.

Shi, H., Verma, M., Zhang, L., Dong, C., Flavell, R. A. and Bennett, A. M. (2013). Improved regenerative myogenesis and muscular dystrophy in mice lacking Mkp5. *J Clin Invest* **123**, 2064-77.

Shimatsu, Y., Katagiri, K., Furuta, T., Nakura, M., Tanioka, Y., Yuasa, K., Tomohiro, M., Kornegay, J. N., Nonaka, I. and Takeda, S. (2003). Canine X-linked muscular dystrophy in Japan (CXMDJ). *Exp Anim* **52**, 93-7.

Shin, J. H., Greer, B., Hakim, C. H., Zhou, Z., Chung, Y. C., Duan, Y., He, Z. and Duan, D. (2013a). Quantitative phenotyping of Duchenne muscular dystrophy dogs by comprehensive gait analysis and overnight activity monitoring. *PLoS One* **8**, e59875.

Shin, J. H., Pan, X., Hakim, C. H., Yang, H. T., Yue, Y., Zhang, K., Terjung, R. L. and Duan, D. (2013b). Microdystrophin ameliorates muscular dystrophy in the canine model of Duchenne muscular dystrophy. *Mol Ther* **21**, 750-7.

Smith, B. F., Kornegay, J. N. and Duan, D. (2007). Independent canine models of Duchenne muscular dystrophy due to intronic insertions of repetitive DNA. *Mol Ther* **15**, S51.

Smith, B. F., Yue, Y., Woods, P. R., Kornegay, J. N., Shin, J. H., Williams, R. R. and Duan, D. (2011). An intronic LINE-1 element insertion in the dystrophin gene aborts dystrophin expression and results in Duchenne-like muscular dystrophy in the corgi breed. *Lab Invest* **91**, 216-31.

Steinberger, M., Foller, M., Vogelgesang, S., Krautwald, M., Landsberger, M., Winkler, C. K., Kasch, J., Fuchtbauer, E. M., Kuhl, D., Voelkl, J. et al. (2014). Lack of the serum- and glucocorticoid-inducible kinase SGK1 improves muscle force characteristics and attenuates fibrosis in dystrophic mdx mouse muscle. *Pflugers Arch*.

Tinsley, J., Deconinck, N., Fisher, R., Kahn, D., Phelps, S., Gillis, J. M. and Davies, K. (1998). Expression of full-length utrophin prevents muscular dystrophy in mdx mice. *Nat Med* **4**, 1441-4.

Valentine, B. A., Cooper, B. J., Cummings, J. F. and deLahunta, A. (1986). Progressive muscular dystrophy in a golden retriever dog: light microscope and ultrastructural features at 4 and 8 months. *Acta Neuropathol (Berl)* **71**, 301-10.

Vallese, D., Negroni, E., Duguez, S., Ferry, A., Trollet, C., Aamiri, A., Vosshenrich, C. A., Fuchtbauer, E. M., Di Santo, J. P., Vitiello, L. et al. (2013). The Rag2(-)Il2rb(-)Dmd(-) mouse: a novel dystrophic and immunodeficient model to assess innovating therapeutic strategies for muscular dystrophies. *Mol Ther* **21**, 1950-7.

van Putten, M., Hulsker, M., Young, C., Nadarajah, V. D., Heemskerk, H., van der Weerd, L., t Hoen, P. A., van Ommen, G. J. and Aartsma-Rus, A. M. (2013). Low dystrophin levels increase survival and improve muscle pathology and function in dystrophin/utrophin double-knockout mice. *FASEB J* **27**, 2484-95.

Vetrone, S. A., Montecino-Rodriguez, E., Kudryashova, E., Kramerova, I., Hoffman, E. P., Liu, S. D., Miceli, M. C. and Spencer, M. J. (2009). Osteopontin promotes fibrosis in dystrophic mouse muscle by modulating immune cell subsets and intramuscular TGF-beta. *J Clin Invest* **119**, 1583-94.

Vidal, B., Ardite, E., Suelves, M., Ruiz-Bonilla, V., Janue, A., Flick, M. J., Degen, J. L., Serrano, A. L. and Munoz-Canoves, P. (2012). Amelioration of Duchenne muscular dystrophy in mdx mice by elimination of matrix-associated fibrin-driven inflammation coupled to the alphaMbeta2 leukocyte integrin receptor. *Hum Mol Genet* **21**, 1989-2004.

Villalta, S. A., Nguyen, H. X., Deng, B., Gotoh, T. and Tidball, J. G. (2009). Shifts in macrophage phenotypes and macrophage competition for arginine metabolism affect the severity of muscle pathology in muscular dystrophy. *Hum Mol Genet* **18**, 482-96.

Wagner, K. R., McPherron, A. C., Winik, N. and Lee, S. J. (2002). Loss of myostatin attenuates severity of muscular dystrophy in mdx mice. *Ann Neurol* **52**, 832-6.

Walmsley, G. L., Arechavala-Gomeza, V., Fernandez-Fuente, M., Burke, M. M., Nagel, N., Holder, A., Stanley, R., Chandler, K., Marks, S. L., Muntoni, F. et al. (2010). A Duchenne muscular dystrophy gene hot spot mutation in dystrophin-deficient cavalier king charles spaniels is amenable to exon 51 skipping. *PLoS One* **5**, e8647.

Walsh, S., Nygren, J., Ponten, A. and Jovinge, S. (2011). Myogenic reprogramming of bone marrow derived cells in a W(4)(1)Dmd(mdx) deficient mouse model. *PLoS One* **6**, e27500.

Wang, B., Li, J., Fu, F. H., Chen, C., Zhu, X., Zhou, L., Jiang, X. and Xiao, X. (2008). Construction and analysis of compact muscle-specific promoters for AAV vectors. *Gene Ther* **15**, 1489-99.

Warner, L. E., DelloRusso, C., Crawford, R. W., Rybakova, I. N., Patel, J. R., Ervasti, J. M. and Chamberlain, J. S. (2002). Expression of Dp260 in muscle tethers the actin cytoskeleton to the dystrophin-glycoprotein complex and partially prevents dystrophy. *Hum Mol Genet* **11**, 1095-105.

Wasala, N. B., Zhang, K., Wasala, L., Hakim, H. C., Duan, D. (2015). The FVB genetic background does not dramatically alter the dystrophic phenotype of mdx mice. *PLoS Curr Muscular Dystrophy*. in-press.

Wein, N., Vulin, A., Falzarano, M. S., Szigyarto, C. A., Maiti, B., Findlay, A., Heller, K. N., Uhlen, M., Bakthavachalu, B., Messina, S. et al. (2014). Translation from a DMD exon 5 IRES results in a functional dystrophin isoform that attenuates dystrophinopathy in humans and mice. *Nat Med* **20**, 992-1000.

Wells, D. J., Wells, K. E., Asante, E. A., Turner, G., Sunada, Y., Campbell, K. P., Walsh, F. S. and Dickson, G. (1995). Expression of human full-length and minidystrophin in transgenic mdx mice: implications for gene therapy of Duchenne muscular dystrophy. *Hum Mol Genet* **4**, 1245-50.

Wells, D. J., Wells, K. E., Walsh, F. S., Davies, K. E., Goldspink, G., Love, D. R., Chan-Thomas, P., Dunckley, M. G., Piper, T. and Dickson, G. (1992). Human dystrophin expression corrects the myopathic phenotype in transgenic mdx mice. *Hum Mol Genet* **1**, 35-40.

Wertz, K. and Fuchtbauer, E. M. (1998). Dmd(mdx-beta geo): a new allele for the mouse dystrophin gene. *Dev Dyn* **212**, 229-41.

Wetterman, C. A., Harkin, K. R., Cash, W. C., Nietfield, J. C. and Shelton, G. D. (2000). Hypertorphic muscular dystrophy in a young dog. *J Am Vet Med Assoc* **216**, 878-81.

Wieczorek, L. A., Garosi, L. S. and Shelton, G. D. (2006). Dystrophin-deficient muscular dystrophy in an old English sheepdog. *Vet Rec* **158**, 270-3.

Winand, N. J., Edwards, M., Pradhan, D., Berian, C. A. and Cooper, B. J. (1994a). Deletion of the dystrophin muscle promoter in feline muscular dystrophy. *Neuromuscul Disord* **4**, 433-45.

Winand, N. J., Pradhan, D. and Cooper, B. J. (1994b). Molecular characterization of severe Duchenne-type muscular dystrophy in a family of Rottwiler dogs. In *Molecular Mechanism of Neuromuscular Disease*. Tucson, Arizona: Muscular Dystrophy Association.

Yang, H. T., Shin, J. H., Hakim, C. H., Pan, X., Terjung, R. L. and Duan, D. (2012). Dystrophin deficiency compromises force production of the extensor carpi ulnaris muscle in the canine model of Duchenne muscular dystrophy. *PLoS One* **7**, e44438.
